# Vitamin B12, folate, and homocysteine in metabolic syndrome: a systematic review and meta-analysis

**DOI:** 10.3389/fendo.2023.1221259

**Published:** 2023-09-13

**Authors:** Juan R. Ulloque-Badaracco, Enrique A. Hernandez-Bustamante, Esteban A. Alarcon-Braga, Ali Al-kassab-Córdova, Juan C. Cabrera-Guzmán, Percy Herrera-Añazco, Vicente A. Benites-Zapata

**Affiliations:** ^1^ Facultad De Ciencias De La Salud, Universidad Peruana De Ciencias Aplicadas, Lima, Peru; ^2^ Sociedad Científica De Estudiantes De Medicina De La Universidad Nacional De Trujillo, Trujillo, Peru; ^3^ Grupo Peruano De Investigación Epidemiológica, Unidad Para La Generación y Síntesis De Evidencias En Salud, Universidad San Ignacio De Loyola, Lima, Peru; ^4^ Centro de Excelencia en Investigaciones Económicas y Sociales en Salud, Universidad San Ignacio de Loyola, Lima, Peru; ^5^ Universidad Privada Del Norte, Trujillo, Peru; ^6^ Red Peruana De Salud Colectiva, Lima, Peru; ^7^ Unidad De Investigación Para La Generación y Síntesis De Evidencias En Salud, Vicerrectorado De Investigación, Universidad San Ignacio De Loyola, Lima, Peru

**Keywords:** metabolic syndrome, folate, vitamin B12, homocysteine, meta-analysis

## Abstract

**Background & aims:**

Metabolic syndrome (MetS) is associated with life-threatening conditions. Several studies have reported an association of vitamin B12, folic acid, or homocysteine (Hcy) levels with MetS. This systematic review and meta-analysis assessed the association of vitamin B12, folic acid, and Hcy levels with MetS.

**Methods:**

PubMed, Scopus, Embase, Ovid/Medline, and Web of Science were searched up to February 13, 2023. Cross-sectional, case-control, or cohort studies were included. A random-effects model was performed using the DerSimonian and Laird method to estimate the between-study variance. Effect measures were expressed as odds ratios (OR) with their corresponding 95% confidence intervals (95% CI). Between-study heterogeneity was evaluated using Cochran’s Q test and the I^2^ statistic.

**Results:**

Sixty-six articles (n = 87,988 patients) were included. Higher vitamin B12 levels were inversely associated with MetS (OR = 0.87; 95% CI: 0.81–0.93; p < 0.01; I^2 ^= 90%). Higher Hcy levels were associated with MetS (OR = 1.19; 95% CI: 1.14–1.24; p < 0.01; I^2 ^= 90%). Folate levels were not associated with MetS (OR = 0.83; 95% CI: 0.66–1.03; p = 0.09; I^2 ^= 90%).

**Conclusion:**

Higher vitamin B12 levels were inversely associated with MetS, whereas higher Hcy levels were associated with MetS. Studies assessing the pathways underlying this association are required.

## 1 Introduction

Metabolic syndrome (MetS) refers to a cluster of interrelated, and often coexisting, disorders such as hyperglycemia, dyslipidemia, hypertension, and abdominal obesity, each of which are independently associated with the risk of developing cardiovascular disease, diabetes, stroke, and increased all-cause mortality (1–5). The incidence of MetS is proportional to the incidence of obesity and type 2 diabetes mellitus; however, the prevalence estimates vary according to the criteria used for the definition of MetS (6). Despite this, studies have shown an increase in its prevalence in recent years. For example, the prevalence of MetS increased from 28.23%–37.09% between 1999 and 2018 in the United States (7), and from 40.2% to 56.31% between 2006 and 2018 in Mexico (8).

In recent years, several studies have investigated the factors affecting MetS, such as physical activity and genetic profile (9, 10). Likewise, the role of some dietary elements in the risk of MetS has also been studied. Some studies have found a lower risk of MetS in patients with a higher intake of fiber, magnesium, selenium, or calcium (11–13). Vitamins have also been suggested to play a role in the risk of MetS. Some studies have found that altered levels of vitamin C, vitamin D, and carotenoids are associated with MetS or some of its components (14–17). Likewise, the association between low levels of folate, vitamin B6, and vitamin B12 or increased homocysteine (Hcy) with the risk of MetS was also suggested because their alterations may also trigger various changes involved in its pathogenesis (18). These three elements are interrelated. Folate is an essential nutrient involved in many crucial functions in the body, and its deficiency is related to an increase in serum Hcy levels. Likewise, vitamin B12 is a cofactor in the synthesis of Hcy from methionine; therefore, deficiency of vitamin B12 can lead to hyperhomocysteinemia (19).

Although not always simultaneously, levels of vitamin B12, folate, and Hcy have been shown to be associated with the risk of stroke, thyroid disease, kidney disease, cardiovascular mortality, and general mortality (20–23). Moreover, there is evidence to suggest that modifying the levels of these macromolecules may prevent some of these outcomes (19). Therefore, evidence of the association between the levels of vitamin B12, folate, and Hcy with MetS would open the possibility of exploring the use of these nutrients as a preventive therapy for MetS or its consequences (24, 25).

Although several primary studies have found an association between levels of vitamin B12, folate, or Hcy and MetS, their results have been inconsistent. To the best of our knowledge, the available evidence has not been systematically reported. Therefore, the objective of this research was to perform a systematic literature review and meta-analysis to synthesize the available evidence of the association between the levels of vitamin B12, folic acid, or Hcy and MetS.

## 2 Methods

### 2.1 Registration and reporting

This study was conducted in line with the methods recommended by the Preferred Reporting Items for Systematic Reviews and Meta-Analyses (PRISMA) statement for reporting the results (26), taking into account the AMSTAR 2 domains (27). A short version of the protocol of this systematic review has been submitted to the International Prospective Register of Systematic Reviews (PROSPERO) [CRD42023402162].

### 2.2 Search Strategy and databases

The search strategy was aligned with the Peer Review of Electronic Search Strategies (PRESS) Checklist (28). It was based on using MeSH, Emtree, and free terms. Subsequently, the search formula was adapted for all databases with no restrictions for date or language. The systematic search was simultaneously run from inception through February 13, 2023, in the following databases: PubMed, Scopus, Embase, Ovid/Medline, and Web of Science. The complete search strategy is presented as **Supplementary Material (Table S1)**.

### 2.3 Study eligibility criteria and data extraction

Cross-sectional, case-control, or cohort studies assessing the associations between vitamin B12, folate, or Hcy and MetS were included. Narrative reviews, scoping reviews, systematic reviews, and conference abstracts were excluded. Four authors independently performed all phases of the study-selection process. All retrieved references were exported from databases to Rayyan© (29). After eliminating duplicate records, four researchers (J.R.U-B, E.A.H-B, E.A.A-B, and J.C.C-G) independently screened the titles and abstracts of each article. After identifying the potential eligible references, the same researchers independently assessed the full-text version of each article. Discrepancies were resolved by consensus. The data extraction was independently performed by this authors using a standardized data extraction sheet built in Google Sheets©. Thereafter, the following information was extracted: first author, publication date, study location, sample size, age, sex, MetS proportion, MetS diagnostic criteria, folate levels (ng/mL), vitamin B12 levels (pg/mL), Hcy levels (µmol/L), and assay methods. The outcome was the association between these markers and MetS.

### 2.4 Risk of bias and publication bias

The quality assessment was independently conducted by two authors (J.R.U-B and A.A-C) using the Newcastle-Ottawa Scale (NOS) for cohort and case-control studies (30), and an adaptation of the NOS for cross-sectional studies (NOS-CS). A score ≥7 stars was considered indicative of a low risk of bias, whereas a score <7 stars was considered indicative of a high risk of bias. Publication bias was assessed using funnel plots, the Begg test, and the trim-and-fill method (31, 32).

### 2.5 Data synthesis

The statistical analysis was performed using STATA 17.0^©^ (Stata Corporation, College Station, TX, USA) and Review Manager v.5.4 (The Cochrane Collaboration, Copenhagen, Denmark). A random-effects model was performed using the DerSimonian and Laird method to estimate the between-study variance. Effect measures were only expressed as odds ratios (OR) with their corresponding 95% confidence intervals (95% CI). Thus, any other measure of effect was transformed into OR. Medians and their interquartile ranges were transformed into means and their corresponding standard deviations using Hozo’s method (33). Chinn’s method was used to transform standard mean differences to their corresponding natural logarithm of the OR (lnOR) and its standard error (34). Between-study heterogeneity was evaluated using Cochran’s Q test and the I^2^ statistic. High heterogeneity was defined as I^2^ ≥ 60% and a p-value < 0.05 in Cochran’s Q test. To identify sources of heterogeneity, subgroup analyses were performed disaggregated by continents, MetS diagnostic criteria, assay method, and sex. In addition, a sensitivity analysis using only studies with a low risk of bias was performed.

## 3 Results

### 3.1 Study selection

**Figure 1:**
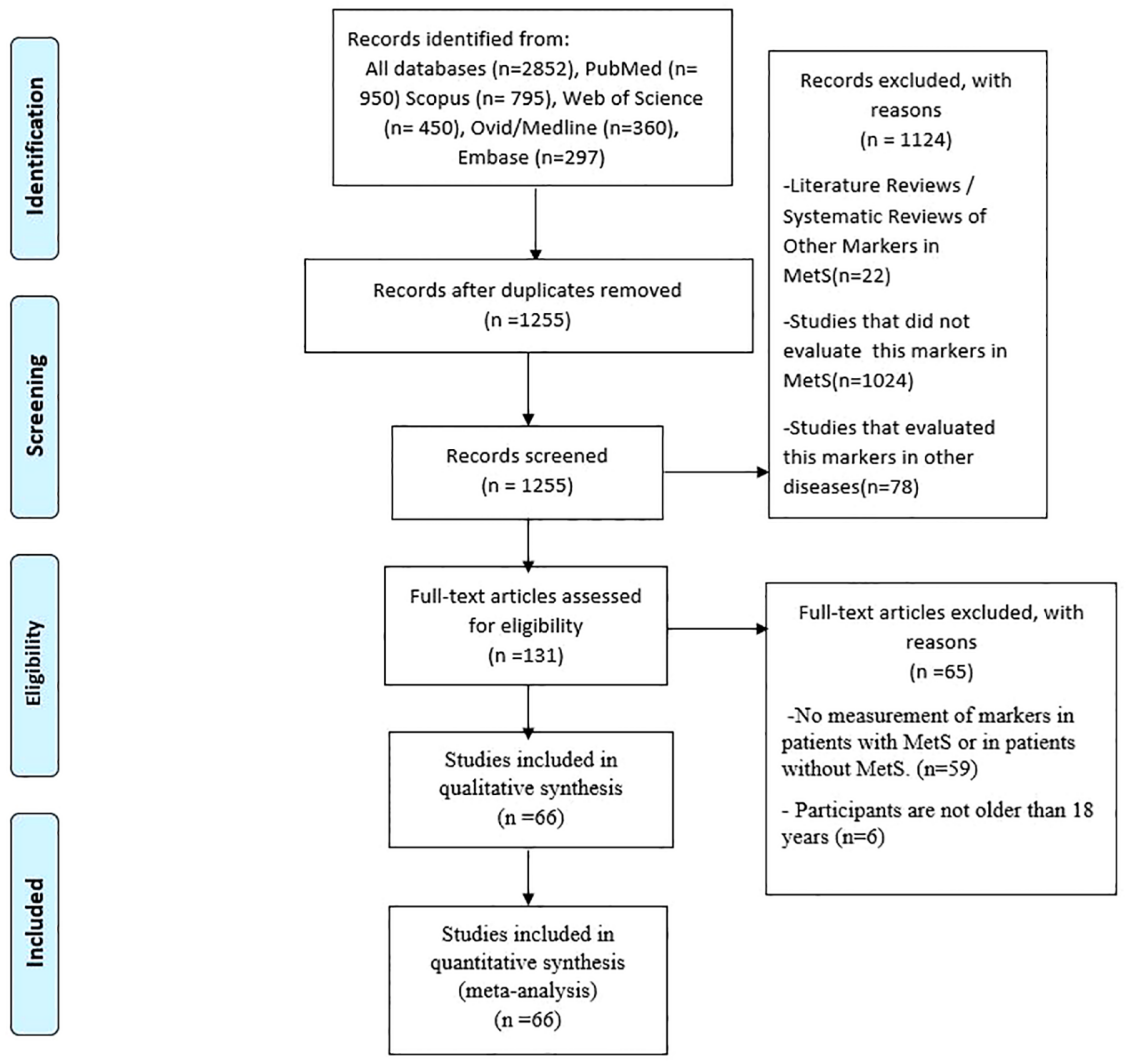
PRISMA Flow Diagram.

The systematic literature search yielded 2,852 records, of which 1,597 were eliminated due to duplication. After screening out articles by abstract-title and full-texts based on the inclusion criteria, 66 articles were identified as eligible for this systematic review and meta-analysis (18, 35–97). A schematic illustration of the literature search and selection is shown in **Figure 1**.

### 3.2 Study characteristics

A total of 66 articles were included, including 9 cohort studies, 48 cross-sectional studies, and 13 case-controls. As four articles (41, 51, 60, 68) had analyzed the association in two different groups of participants each, a total of 70 records were included. The geographic distribution was as follows: China (11 articles), South Korea (9 articles), Turkey (7 articles), United States (6 articles), Italy (3 articles), Iran (3 articles), India (3 articles), Serbia (2 articles), Bangladesh (2 articles), France (2 articles), Saudi Arabia (2 articles), Spain (1 article), Ecuador (1 article), Netherlands (1 article), Canada (1 article), Taiwan (1 article), Australia (1 article), Romania (1 article), Trinidad & Tobago (1 article), Nigeria (1 article), Poland (1 article), Greece (1 article), South Africa (1 article), Slovenia (1 article), Cameroon (1 article), Peru (1 article), and multiple Mesoamerican countries (1 article). A total of 87,988 patients (42,279 male and 35,727 female) were evaluated. Six studies (n = 9,982) did not report the sex of the included participants. These characteristics are summarized in **Supplementary Table S2**.

The criteria for diagnosing MetS in the included studies were those recommended by the American Heart Association/National Heart Lung and Blood Institute (AHA/NHLBI) (98), Chinese Diabetes Society (CDS) (99), National Cholesterol Education Program-Adult Treatment Panel III (NCEP-ATP III) (100), International Diabetes Federation (IDF) (101), and the World Health Organization (WHO 1999) (102). The definitions of MetS used for each criterion are presented in **Supplementary Table S3**.

The methods used to measure Hcy, vitamin B12, and folate levels were chemiluminescence immunoassay (CLIA), electrochemiluminescence immunoassay (ECLIA), enzyme-linked immunosorbent assay (ELISA), fluorescence polarization immunoassay (FPI), high-performance liquid chromatography (HPLC), nephelometry immunoassay, radioimmunoassay, homogeneous enzyme immunoassay, and competitive displacement assay.

After the risk of bias assessment using the NOS, a total of 63 studies were classified as low risk of bias, and 7 studies as high risk of bias (**Supplementary Table S4**).

### 3.3 Association between vitamin B12 levels and MetS

**Figure 2:**
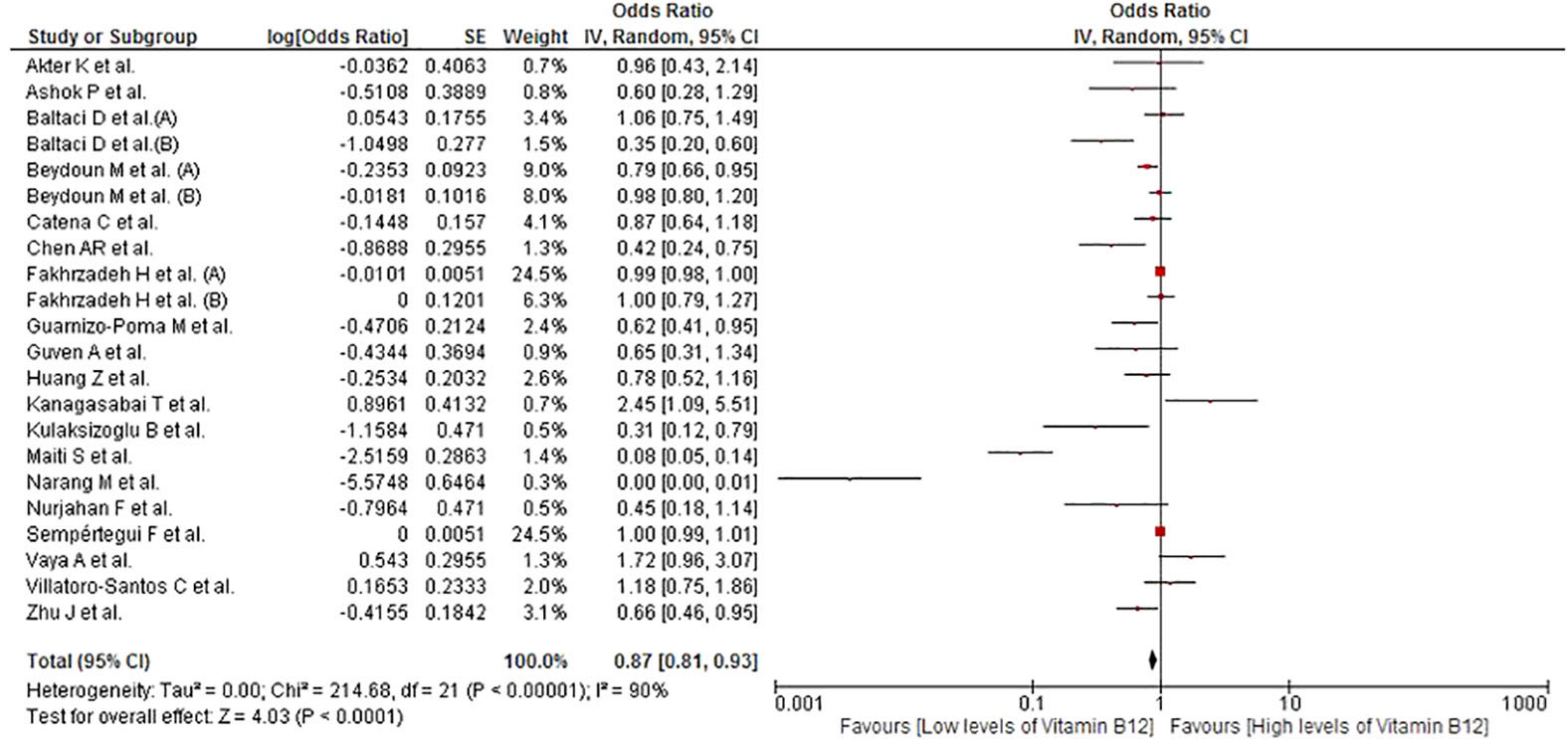
Association between vitamin B12 and MetS.

This association was evaluated in 22 studies involving a total of 15,115 participants. Higher vitamin B12 levels were inversely associated with MetS (OR = 0.87; 95% CI: 0.81–0.93; p < 0.01; I^2 ^= 90%) (**Figure 2**). Subgroup analyses were performed according to continents (**Supplementary Figure S1**), sex (**Supplementary Figure S2**), assay method (**Supplementary Figure S3**), and MetS diagnostic criteria (**Supplementary Figure S4**). Studies conducted in Asian countries (OR = 0.37; 95% CI: 0.22–0.64; p < 0.01; I^2 ^= 95%), those using ECLIA to measure B12 levels (OR = 0.15; 95% CI: 0.02–0.85; p < 0.01; I^2 ^= 97%), and those using the AHA/NHLBI criteria for MetS diagnosis (OR = 0.25; 95% CI: 0.09–0.72; p < 0.01; I^2 ^= 96%) showed a consistent association with no decrease in heterogeneity. In the sensitivity analysis (**Supplementary Figure S5**), after eliminating studies at high risk of bias, the association persisted (OR = 0.62; 95% CI: 0.48–0.79; p < 0.01) and heterogeneity was high (I^2 ^= 92%).

### 3.4 Association between folate levels and MetS

**Figure 3:**
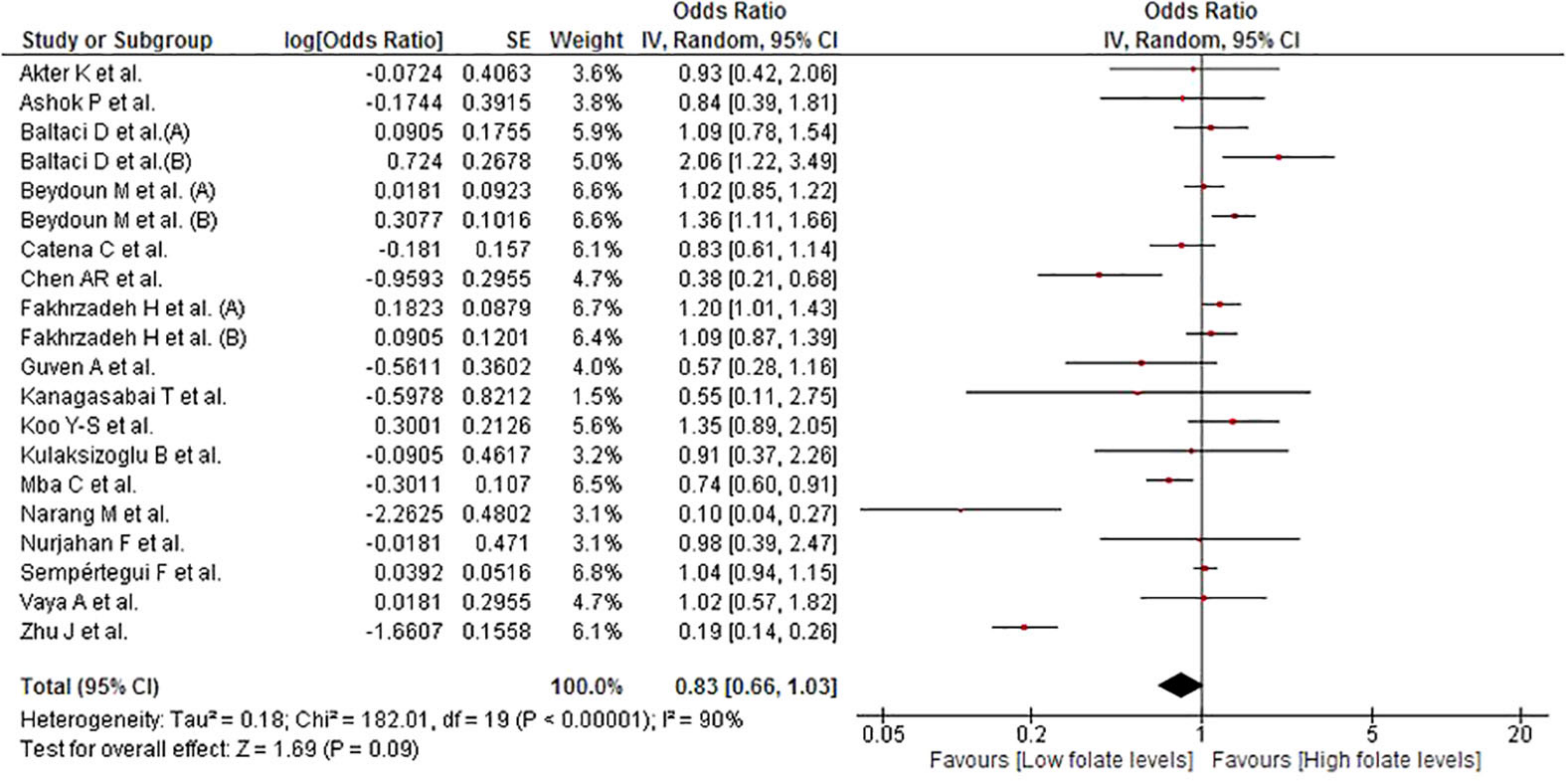
Association between folate and MetS.

This association was evaluated in 20 studies involving a total of 15,869 participants. No significant association was found between folate levels and MetS (OR = 0.83; 95% CI: 0.66–1.03; p = 0.09; I^2 ^= 90%) (**Figure 3**). Given the high heterogeneity, subgroup analyses by continent (**Supplementary Figure S6**), sex (**Supplementary Figure S7**), assay method (**Supplementary Figure S8**), and MetS diagnostic criteria (**Supplementary Figure S9**) were performed. In the subgroup of women, higher folate levels were significantly associated with the risk of MetS (OR = 1.35; 95% CI: 1.13–1.60; p < 0.01; I^2 ^= 5%). In the subgroup of AHA/NHLBI diagnostic criteria, higher folate levels were inversely associated with MetS (OR = 0.36; 95% CI: 0.16–0.81; p = 0.01; I^2 ^= 96%). In the sensitivity analysis (**Supplementary Figure S10**), after removing studies at high risk of bias, there was no significant association and the heterogeneity remained high (OR = 0.78; 95% CI: 0.59–1.03; p = 0.08; I^2 ^= 92%).

### 3.5 Association between homocysteine levels and MetS

**Figure 4:**
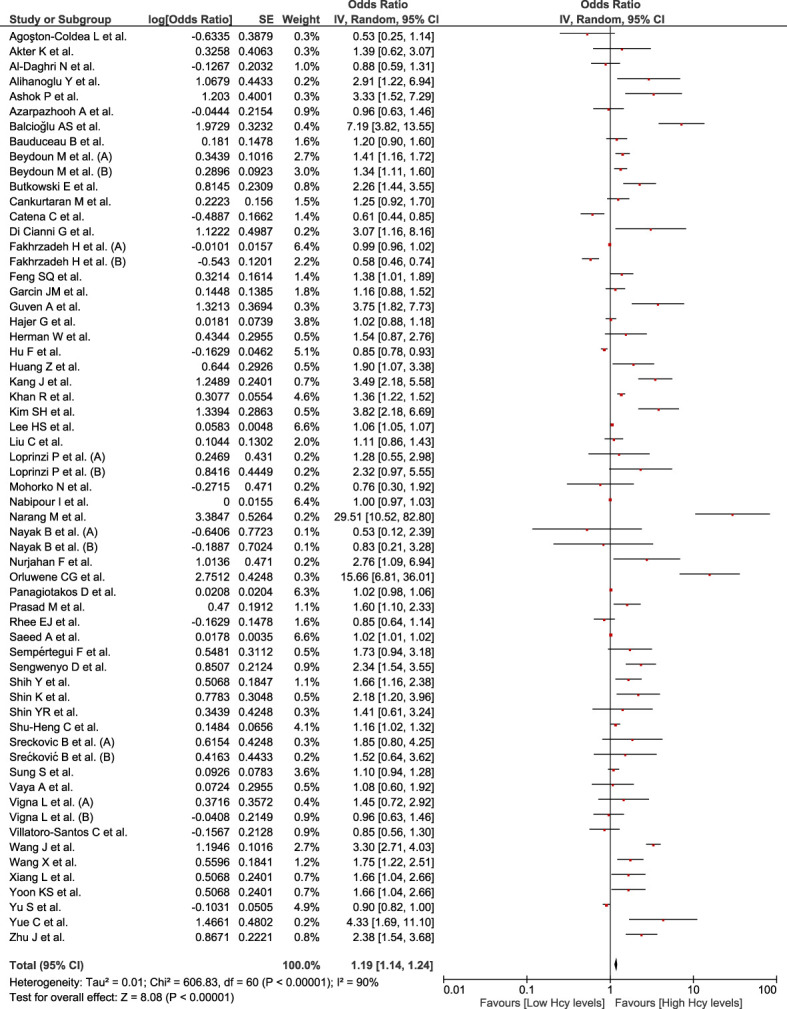
Association between homocysteine and Mets.

This association was evaluated in 61 studies involving a total of 81,888 participants. Higher Hcy levels were associated with MetS (OR = 1.19; 95% CI: 1.14–1.24; p < 0.01; I^2 ^= 90%) (**Figure 4**). Owing to the high heterogeneity, subgroup analysis according to continents (**Supplementary Figure S11**), sex (**Supplementary Figure S12**), assay method (**Supplementary Figure S13**), and MetS diagnostic criteria (**Supplementary Figure S14**) were conducted. All the subgroups exhibited statistically significant associations, except for the subgroup that used the HPLC method to quantify the Hcy levels. On sensitivity analysis (**Supplementary Figure S15**), the association remained significant after eliminating the studies at high risk of bias (OR = 1.21; 95% CI: 1.16–1.26; p < 0.01); yet, there was high heterogeneity (I^2 ^= 90%).

### 3.6 Publication bias

Begg test revealed no significant influence of publication bias on the association of vitamin B12 and folate with MetS (Begg test <0.1). Nonetheless, the association between Hcy and MetS was corrected using the trim-and-fill method (OR = 1.031; 95% CI: 1.025–1.036) (**Supplementary Figure S16**).

## 4 Discussion

This systematic review and subsequent meta-analysis separately evaluated the association between vitamin B12, folate, or Hcy level and MetS. Higher vitamin B12 levels were inversely associated with MetS. Contrarily, higher Hcy levels were associated with MetS. The first association was driven mostly by studies from the Asian continent, while the latter showed no differences in the subgroup analyses. On the other hand, there was no association between folate levels and MetS; however, on subgroup analysis, folate levels showed an association with MetS in women. Overall, this systematic review and meta-analysis may facilitate an in-depth understanding of these associations.

The interaction between vitamin B12 and MetS is complex and not completely understood. Studies have shown that deficiency of folate, vitamin B6, and vitamin B12 can cause dyslipidemia, vascular endothelial dysfunction, glucose intolerance, and insulin resistance through oxidative stress (18). Their deficiency leads to systemic inflammation and impaired nitric oxide synthesis, all of which are implicated in the pathophysiology of MetS (18). Vitamin B12 is an essential hydrosoluble vitamin that plays an important role in DNA methylation as well as amino acid and lipid homeostasis through the regulation of one-carbon metabolism (25). Low levels of vitamin B12 can interfere with DNA synthesis and cellular inflammation, and increase fat and Hcy synthesis (25).

An increase in the binding of vitamin B12 to haptocorrins secondary to their increased plasma levels, especially for transcobalamin (TCB) I and III, which are the main haptocorrins, leads to a potential decline in their attachment to TCB II and, therefore, alters their delivery to cells (103). This phenomenon can cause a functional vitamin B12 deficiency with an increase in Hcy and/or methylmalonic acid levels, even though the initial abnormality is not a vitamin B12 deficiency (103). This is relevant, since vitamin B12 is essential for facilitating energy, lipid, and carbohydrate metabolism in humans, mainly by counteracting hyperhomocysteinemia (18, 104).

Mammalian cells exhibit two different vitamin B12-dependent reactions: the conversion of methylmalonyl coenzyme A to succinyl coenzyme and the methylation of Hcy to methionine (25). Therefore, vitamin B12 deficiency results in elevated serum levels of Hcy and methylmalonic acid, which serve as biochemical markers of vitamin B12 status. Therefore, methylmalonic acid accumulates in patients with B12 deficiency, which is associated with lipogenesis and insulin resistance (25). Although there are multiple pathogenic mechanisms of MetS, insulin resistance is one of the most important underlying causes.

The underlying mechanism of the association between insulin resistance and the MetS is not fully understood, but some metabolic pathways have been proposed. Insulin resistance causes an increase in circulating glucose leading to increased insulin secretion (25). Consequently, glucose transporter types 1-3 (GLUT1-3), which are insulin-independent and found in neurons, renal cells, and red blood cells, are exposed to large amounts of glucose, leading to glucotoxicity (105). Persistent insulin resistance exhausts the ability of pancreatic beta cells to provide the required amounts of insulin, further disrupting glucose homeostasis (106). Additionally, there is an increase in the synthesis of triglycerides and very low-density lipoproteins that contribute to the failure of the GLUT4 transporter (106, 107). According to the portal theory of MetS, free fatty acids are released from the accumulated visceral fat (25). These free fatty acids affect the activity of phosphoinositide-3-kinase, reducing its function and aggravating insulin resistance (106). In addition, they contribute to endothelial damage by producing reactive oxygen species, along with the hyperinsulinemia state and cytokines produced by adipose tissue (25).

Despite this plausibility, there are discrepancies on whether levels of vitamin B12 or a diet rich in vitamin B12 reduce the risk of MetS as opposed to other components of the vitamin B complex. These discrepancies may be partially attributable to differences between the studies with respect to methodology, clinical features, and ethnic composition of the study population (18). Although this study does not solve these questions, it provides evidence of the potential linkage between vitamin B12 levels and MetS (18). Therefore, randomized studies assessing the potential beneficial effect of vitamin B12 supplementation are needed. Long-term vitamin B12 supplementation has been shown to reduce weight gain in overweight or obese people (19). In the present study, a stronger association was observed in the studies conducted in the Asian continent, which suggests that ethnic, dietetic, or genetic factors may influence this association (108, 109). Hence, future studies should control for these potential effect modifiers. It is necessary to emphasize that a large percentage of studies included in this review were conducted in Asian countries, where deficiencies of other micronutrients, such as vitamin A or D, have also been described (110, 111). These micronutrient deficiencies were also shown to be associated with MetS (112, 113). Thus, our systematic review does not allow us to discern whether our findings, despite being biologically plausible, are independent of these associations.

We observed no association between endogenous folate levels and MetS, which is not consistent with some studies of folate supplementation. Nevertheless, in the subgroup analysis by sex, folate deficiency showed an association with MetS among women. Sex-based differences in the effect of progression of metabolic risk factors on the development of diabetes have been reported (114). Further studies are required to unravel the underlying causes of this difference. On the other hand, folic acid supplementation has been shown toimprove clinical and laboratory outcomes. In a randomized clinical trial enrolling obese women with polycystic ovary syndrome, folic acid supplementation improved metabolic profiles (115). Similarly, in another trial, both men and women exhibited improved insulin resistance and endothelial dysfunction as well as decreasing Hcy levels after treatment with folic acid and vitamin B12 (116). A systematic review of randomized trials found beneficial effects of folic acid on inflammatory markers–such as hs-CRP, IL-6, and TNF-α–in patients with MetS. Indeed, low levels of folate, like vitamin B12, can interfere with DNA synthesis, increase cellular inflammation, and elevate fat and Hcy synthesis (25).

Several studies have documented the association between Hcy and MetS (62, 64), and Hcy has even been proposed as a marker (40). Increased levels of Hcy were shown to be associated with cardiovascular death (64), stroke (117), and obesity (118). Nonetheless, it is important to note that the high levels of Hcy in patients with MetS could be iatrogenic, as most hypolipidemic, antidiabetic, and antihypertensive drugs raise circulating Hcy levels (119). The biological plausibility of these associations relies on various biological effects secondary to hyperhomocysteinemia, such as vascular damage, oxidative stress-induced DNA damage, neuronal apoptosis, cellular cytotoxicity, and endothelial nitric oxide production, which could trigger MetS (55, 120). Moreover, since Hcy metabolism is regulated by vitamin B6, folate, and vitamin B12, the effect of Hcy may also be partly mediated by variations in these vitamins (19). On the other hand, the reported sex-based differences are not fully understood but some authors hypothesize that estrogen status is inversely associated with total circulating Hcy level, independent of nutritional status and muscle mass (121). Dietary factors may also explain the differences reported in subgroup analysis by continent (122).

The differences in the levels of the macromolecules studied suggest the potential therapeutic role of supplementation of these macromolecules. Several clinical trials and observational studies have studied folic acid and vitamin B12 supplementation for the management of MetS and control of Hcy levels (18, 123, 124), as well as other cardiovascular outcomes (115, 125), with apparently positive results. Thus, this evidence needs to be synthesized in future studies.

### 4.1 Limitations

Some limitations of this study should be considered while interpreting the findings. First, the lack of data prevented a meta-analysis of the sensitivity, specificity, and optimal cut-off points of these variables. Moreover, we did not compare the pooled mean levels of the macromolecules between participants with and without MetS. Second, high statistical heterogeneity was found due to methodological and clinical differences between studies, which only decreased in some subgroups. Third, the studies had not adjusted the marker levels for potential confounding factors, such as lifestyle, diet quality, sociodemographic conditions, and comorbidities. Given the observational nature of the included studies, the study findings should be interpreted as exploratory. Fourth, a large proportion of studies included in our meta-analysis were conducted in Asia; thus, it would be worthwhile to conduct further research on this topic in other geographic regions. Fifth, given that most of the included studies were of cross-sectional design and the nature of the exposure and outcome variable, there is a risk of retro-causality. Nonetheless, this study has several strengths. A large number of studies and participants were included. Moreover, several subgroups were analyzed. To the best of our knowledge, this is the first systematic review and meta-analysis assessing the association between vitamin B12, Hcy, or folate and MetS.

## 5 Conclusion

Higher vitamin B12 levels were inversely associated with MetS. Further studies are required to assess the pathways underlying this association.

## Author contributions

JU-B: Conceptualization, Methodology, Investigation, Formal analysis, and Writing - Original Draft. EH-B: Methodology, Investigation, Formal analysis, and Writing - Original Draft. EA-B: Methodology, Investigation, Formal analysis, and Writing - Original Draft. AA-k-C: Methodology, Investigation, Formal analysis, and Writing - Original Draft. JC-G: Methodology, Investigation, Formal analysis, and Writing - Original Draft. PH-A: Investigation, Writing - Original Draft, Visualization and Supervision. VB-Z: Conceptualization, Methodology, Writing - Review & Editing, Visualization, and Supervision. All authors contributed to the article and approved the submitted version.
