# Supplementary material for: Vitamin B12, folate, and homocysteine in metabolic syndrome: a systematic review and meta-analysis
Source: Front Endocrinol (Lausanne). 2023 Sep 13;14:1221259. doi: 10.3389/fendo.2023.1221259 (PMC10527372; doi:10.3389/fendo.2023.1221259)

Supplementary Material

Vitamin B12, folate, and homocysteine in metabolic syndrome: A systematic review and meta-analysis

Juan R. Ulloque-Badaracco, Enrique A. Hernandez-Bustamante, Esteban A. Alarcon-Braga, Ali Al-kassab-Córdova, Juan C. Cabrera-Guzmán, Percy Herrera.Añazco, Vicente A. Benites-Zapata

*** Correspondence:** Corresponding Author: vbenites@usil.edu.pe

**Table S1. Search strategies**

| **Source** | **PubMed** |
| --- | --- |
| **Search** | **Formula** |
| **#1** | Metabolic Syndrome [MH] OR Cardiometabolic Syndrome [MH] OR Metabolic Syndrome X [MH] OR Reaven Syndrome X [MH] OR Insulin Resistance Syndrome X [MH] |
| **#2** | Vitamin B 12 [MH] OR (“vitamin*” [TIAB] AND “B12” [TIAB]) OR (“b” [TIAB] AND “12” [TIAB]) OR “cyanocobalamin*” [TIAB] OR “cobalamin*” [TIAB] OR “eritron*” [TIAB] |
| **#3** | Folic Acid [MH] OR ((“Folic” [TIAB] OR “Pteroylglutamic” [TIAB]) AND “Acid” [TIAB]) OR (“Vitamin” [TIAB] AND (“M” [TIAB] OR “B9” [TIAB])) OR “Folvite” [TIAB] OR “Folacin” [TIAB] OR “Folate” [TIAB] |
| **#4** | Homocysteine [MH] OR “2-amino-4-mercaptobutyric acid” [TIAB] OR “2 amino 4 mercaptobutyric acid” [TIAB] OR “homocysteine” [TIAB] OR “2 amino 4 mercaptobutyric acid” [TIAB] OR “betahomocystein” [TIAB] |
| **#5** | #2 OR #3 OR #4 |
| **#6** | #1 AND #5 |
| **Source** | **Scopus** |
| **Search** | **Formula** |
| **#1** | TITLE-ABS-KEY(“Metabolic Syndrome*” OR “Cardiometabolic Syndrome*” OR “Metabolic Syndrome X*” OR “Insulin Resistance Syndrome X*” OR “Reaven Syndrome X*”) |
| **#2** | TITLE-ABS-KEY ((“vitamin*” W/3 “B12”) OR (“b” W/3 “12”) OR “cyanocobalamin*” OR “cobalamin*” OR “eritron*”) |
| **#3** | TITLE-ABS-KEY(((“Folic” OR “Pteroylglutamic”) W/3 “Acid”) OR (“Vitamin” W/3 (“M” OR “B9”)) OR “Folvite” OR “Folacin” OR “Folate”) |
| **#4** | TITLE-ABS-KEY(“2-amino-4-mercaptobutyric acid” OR “2 amino 4 mercaptobutyric acid” OR “homocysteine” OR “2 amino 4 mercaptobutyric acid” OR “betahomocystein”) |
| **#5** | #2 OR #3 OR #4 |
| **#6** | #1 AND #5 |
| **Source** | **Web of Science** |
| **Search** | **Formula** |
| **#1** | TI=(“Metabolic Syndrome*” OR “Cardiometabolic Syndrome*” OR “Metabolic Syndrome X*” OR “Insulin Resistance Syndrome X*” OR “Reaven Syndrome X*”) |
| **#2** | TI=((“vitamin*” NEAR/3 “B12”) OR (“b” NEAR/3 “12”) OR “cyanocobalamin*” OR “cobalamin*” OR “eritron*”) OR AB=((“vitamin*” NEAR/3 “B12”) OR (“b” NEAR/3 “12”) OR “cyanocobalamin*” OR “cobalamin*” OR “eritron*”) OR AK=((“vitamin*” NEAR/3 “B12”) OR (“b” NEAR/3 “12”) OR “cyanocobalamin*” OR “cobalamin*” OR “eritron*”) OR KP=((“vitamin*” NEAR/3 “B12”) OR (“b” NEAR/3 “12”) OR “cyanocobalamin*” OR “cobalamin*” OR “eritron*”) OR TS=((“vitamin*” NEAR/3 “B12”) OR (“b” NEAR/3 “12”) OR “cyanocobalamin*” OR “cobalamin*” OR “eritron*”) |
| **#3** | TI=(((“Folic” OR “Pteroylglutamic”) NEAR/3 “Acid”) OR (“Vitamin” NEAR/3 (“M” OR “B9”)) OR “Folvite” OR “Folacin” OR “Folate”) OR AB=(((“Folic” OR “Pteroylglutamic”) NEAR/3 “Acid”) OR (“Vitamin” NEAR/3 (“M” OR “B9”)) OR “Folvite” OR “Folacin” OR “Folate”) OR KP=(((“Folic” OR “Pteroylglutamic”) NEAR/3 “Acid”) OR (“Vitamin” NEAR/3 (“M” OR “B9”)) OR “Folvite” OR “Folacin” OR “Folate”) OR AK=(((“Folic” OR “Pteroylglutamic”) NEAR/3 “Acid”) OR (“Vitamin” NEAR/3 (“M” OR “B9”)) OR “Folvite” OR “Folacin” OR “Folate”) OR TS=(((“Folic” OR “Pteroylglutamic”) NEAR/3 “Acid”) OR (“Vitamin” NEAR/3 (“M” OR “B9”)) OR “Folvite” OR “Folacin” OR “Folate”) |
| **#4** | TI=(“2-amino-4-mercaptobutyric acid” OR “2 amino 4 mercaptobutyric acid” OR “homocysteine” OR “2 amino 4 mercaptobutyric acid” OR “betahomocystein”) OR AB=(“2-amino-4-mercaptobutyric acid” OR “2 amino 4 mercaptobutyric acid” OR “homocysteine” OR “2 amino 4 mercaptobutyric acid” OR “betahomocystein”) OR AK=(“2-amino-4-mercaptobutyric acid” OR “2 amino 4 mercaptobutyric acid” OR “homocysteine” OR “2 amino 4 mercaptobutyric acid” OR “betahomocystein”) OR KP=(“2-amino-4-mercaptobutyric acid” OR “2 amino 4 mercaptobutyric acid” OR “homocysteine” OR “2 amino 4 mercaptobutyric acid” OR “betahomocystein”) OR TS=(“2-amino-4-mercaptobutyric acid” OR “2 amino 4 mercaptobutyric acid” OR “homocysteine” OR “2 amino 4 mercaptobutyric acid” OR “betahomocystein”) |
| **#5** | #2 OR #3 OR #4 |
| **#6** | #1 AND #5 |
| **Source** | **Embase** |
| **Search** | **Formula** |
| **#1** | 'Metabolic Syndrome'/exp OR (“Cardiometabolic Syndrome*” OR “Metabolic Syndrome X*” OR “Insulin Resistance Syndrome X *” OR “Reaven Syndrome X*”):ti):kw |
| **#2** | 'cyanocobalamin'/exp OR ((“vitamin*” NEAR/3 “B12”) OR (“b” NEAR/3 “12”) OR “cyanocobalamin*” OR “cobalamin*” OR “eritron*”):ti OR ((“vitamin*” NEAR/3 “B12”) OR (“b” NEAR/3 “12”) OR “cyanocobalamin*” OR “cobalamin*” OR “eritron*”):ab OR ((“vitamin*” NEAR/3 “B12”) OR (“b” NEAR/3 “12”) OR “cyanocobalamin*” OR “cobalamin*” OR “eritron*”):kw |
| **#3** | 'folic acid'/exp OR (((“Folic” OR “Pteroylglutamic”) NEAR/3 “Acid”) OR (“Vitamin” NEAR/3 (“M” OR “B9”)) OR “Folvite” OR “Folacin” OR “Folate”):ti OR (((“Folic” OR “Pteroylglutamic”) NEAR/3 “Acid”) OR (“Vitamin” NEAR/3 (“M” OR “B9”)) OR “Folvite” OR “Folacin” OR “Folate”):ab OR (((“Folic” OR “Pteroylglutamic”) NEAR/3 “Acid”) OR (“Vitamin” NEAR/3 (“M” OR “B9”)) OR “Folvite” OR “Folacin” OR “Folate”):kw |
| **#4** | 'homocysteine'/exp OR (“2-amino-4-mercaptobutyric acid” OR “2 amino 4 mercaptobutyric acid” OR “homocysteine” OR “2 amino 4 mercaptobutyric acid” OR “betahomocystein”):ti OR (“2-amino-4-mercaptobutyric acid” OR “2 amino 4 mercaptobutyric acid” OR “homocysteine” OR “2 amino 4 mercaptobutyric acid” OR “betahomocystein”):ab OR (“2-amino-4-mercaptobutyric acid” OR “2 amino 4 mercaptobutyric acid” OR “homocysteine” OR “2 amino 4 mercaptobutyric acid” OR “betahomocystein”):kw |
| **#5** | #2 OR #3 OR #4 |
| **#6** | #1 AND #5 |
| **Source** | **OVID** |
| **Search** | **Formula** |
| **#1** | ('Metabolic Syndrome* OR Cardiometabolic Syndrome* OR Metabolic Syndrome X* OR Insulin Resistance Syndrome X* OR Reaven Syndrome X *).kw. |
| **#2** | ((vitamin* adj3 B12) OR (b adj3 12) OR cyanocobalamin* OR cobalamin* OR eritron*).ti. OR ((vitamin* adj3 B12) OR (b adj3 12) OR cyanocobalamin* OR cobalamin* OR eritron*).ab. OR ((vitamin* adj3 B12) OR (b adj3 12) OR cyanocobalamin* OR cobalamin* OR eritron*).kw. |
| **#3** | (((Folic OR Pteroylglutamic) adj3 Acid) OR (Vitamin adj3 (M OR B9)) OR Folvite OR Folacin OR Folate).ti. OR (((Folic OR Pteroylglutamic) adj3 Acid) OR (Vitamin adj3 (M OR B9)) OR Folvite OR Folacin OR Folate).ab. OR (((Folic OR Pteroylglutamic) adj3 Acid) OR (Vitamin adj3 (M OR B9)) OR Folvite OR Folacin OR Folate).kw. |
| **#4** | (2-amino-4-mercaptobutyric acid OR 2 amino 4 mercaptobutyric acid OR homocysteine OR 2 amino 4 mercaptobutyric acid OR betahomocystein).ti. OR (2-amino-4-mercaptobutyric acid OR 2 amino 4 mercaptobutyric acid OR homocysteine OR 2 amino 4 mercaptobutyric acid OR betahomocystein).ab. OR (2-amino-4-mercaptobutyric acid OR 2 amino 4 mercaptobutyric acid OR homocysteine OR 2 amino 4 mercaptobutyric acid OR betahomocystein).kw. |
| **#5** | #2 OR #3 OR #4 |
| **#6** | #1 AND #5 |

**Table S2. Characteristics of the included studies**

| Author | Year | Country | Median/mean/Range age (IQR/SD) | Participants (male/female) | MetS definition criteria | Marker analyzed | Marker mean (SD) in patients with MetS | Marker mean (SD) in patients without MetS | Assay method | Odds ratio (95% CI) |
| --- | --- | --- | --- | --- | --- | --- | --- | --- | --- | --- |
| Zhu J et al. | 2023 | United States | 24.9(3.6) | 4414(2083/2331) | AHA/NHLBI | Hcy | NR | NR | FPI | 2.38(1.54–3.67) |
|  |  |  |  |  |  | Folate | NR | NR | HEI | 0.19(0.14–0.25) |
|  |  |  |  |  |  | Vitamin B12 | NR | NR |  | 0.66(0.46–0.94) |
| Narang M et al. | 2015 | India | 48.6(11.5) | 75(43/32) | AHA/NHLBI | Hcy | 16.768(6.6) | 6.484(0.873) | CLIA | NR |
|  |  |  |  |  |  | Folate | 3.254(1.92) | 5.312(0.754) | ECLIA | NR |
|  |  |  |  |  |  | Vitamin B12 | 183.704(36.951) | 346.36(74.353) |  | NR |
| Guven A et al. | 2005 | Turkey | 38(25–48) | 101(49/52) | NCEP-ATP III | Hcy | 28.4(23.25) | 14.8(11.85) | FPI | NR |
|  |  |  |  |  |  | Folate | 5.55(4.14) | 8.34(12.17) | CLIA | NR |
|  |  |  |  |  |  | Vitamin B12 | 216.25(162.22) | 257.25(171.11) |  | NR |
| Catena C et al. | 2015 | Italy | 56(13) | 562(301/261) | AHA/NHLBI | Hcy | 11.55(3.85) | 12.62(3.95) | Nephelometry Immunoassay | NR |
|  |  |  |  |  |  | Folate | 5.16(2.69) | 5.43(2.51) | Competitive displacement assay | NR |
|  |  |  |  |  |  | Vitamin B12 | 388.25(153.33) | 401.25(172.59) |  | NR |
| Vaya A et al. | 2011 | Spain | 51(11) | 159(100/59) | NCEP-ATP III | Hcy | 12.03 (3.18) | 11.91(3.53) | FPI | NR |
|  |  |  |  |  |  | Folate | 8.45 (3.37) | 8.43(3.67) | CLIA | NR |
|  |  |  |  |  |  | Vitamin B12 | 584 (252) | 515(218) |  | NR |
| Sempértegui F et al. | 2011 | Ecuador | 74.4(6.4) | 352(127/225) | IDF | Hcy | NR | NR | FPI | 1.73(0.94–3.18) |
|  |  |  |  |  |  | Folate | NR | NR | CLIA | 1.04(0.94–1.15) |
|  |  |  |  |  |  | Vitamin B12 | NR | NR |  | 1(0.99–1.01) |
| Sreckovic B et al. (A) | 2017 | Serbia | 30–75 | 76(NR/NR) | NCEP-ATP III | Hcy | 12.99(3.22) | 11.8(3.74) | FPI | NR |
| Sreckovic B et al. (B) | 2018 | Serbia | 30–66 | 69(NR/NR) | NCEP-ATP III | Hcy | 13.38(2.86) | 12.69(3.1) | CLIA | NR |
| Loprinzi P et al. (A) | 2012 | United States of America | 47.5 (1) | 611(611/0) | NCEP-ATP III | Hcy | NR | NR | FPI | 1.28 (0.55–2.97) |
| Loprinzi P et al. (B) | 2012 | United States of America | 49.3 (0.9) | 535(0/535) | NCEP-ATP III | Hcy | NR | NR | FPI | 2.32 (0.97–5.54) |
| Fakhrzadeh H et al. (A) | 2009 | Iran | 51(10) | 226(61/165) | NCEP-ATP III | Hcy | NR | NR | HPLC | 0.99(0.96–1.02) |
|  |  |  |  |  |  | Folate | NR | NR | RIA | 1.2(1.01–1.42) |
|  |  |  |  |  |  | Vitamin B12 | NR | NR |  | 0.99 (0.98–1.00) |
| Fakhrzadeh H et al. (B) | 2004 | Iran | 41.35(12.2) | 1145(435/710) | NCEP-ATP III | Hcy | 15.64(1.58) | 16.11(1.56) | HPLC | NR |
|  |  |  |  |  |  | Folate | 3.97(1.69) | 3.89(1.63) | RIA | NR |
|  |  |  |  |  |  | Vitamin B12 | 355.6(2.47) | 355.6(2.37) |  | NR |
| Nurjahan F et al. | 2021 | Bangladesh | 25–45 | 60(0/60) | IDF | Hcy | 10.89(4.01) | 8.91(2.85) | CLIA | NR |
|  |  |  |  |  |  | Folate | 8.77(2.29) | 8.81(3.9) |  | NR |
|  |  |  |  |  |  | Vitamin B12 | 396.9(150.52) | 462.5(143.17) |  | NR |
| Akter K et al. | 2021 | Bangladesh | 37.98(6.71) | 80(0/80) | IDF | Hcy | 8.03(3.27) | 7.53(2.17) | CLIA | NR |
|  |  |  |  |  |  | Folate | 8.3(3.12) | 8.42(3.09) |  | NR |
|  |  |  |  |  |  | Vitamin B12 | 409.98(152.88) | 412.78(138.28) |  | NR |
| Hajer G et al. | 2007 | Netherlands | 62.8(10.4) | 2169(1689/480) | NCEP-ATP III | Hcy | 14.5(6.23) | 14.4(7.17) | HPLC | NR |
| Azarpazhooh A et al. | 2020 | Canada | 64.9(14.7) | 972(481/491) | IDF | Hcy | NR | NR | FPI | 0.95(0.62–1.45) |
| Kang J et al. | 2012 | South Korea | 52.17(3.4) | 290(290/0) | NCEP-ATP III | Hcy | 13.11(3.99) | 10.89(2.86) | ELISA | NR |
| Wang J et al. | 2018 | China | 51.23(11.5) | 1378(867/511) | AHA/NHLBI | Hcy | 12.96(4.32) | 10.44(3.27) | CLIA | NR |
| Beydoun M et al. (A) | 2011 | United States of America | 44.6(0.6) | 1597(1597/0) | NCEP-ATP III | Hcy | 9.5(4.62) | 8.8(3.26) | CLIA | NR |
|  |  |  |  |  |  | Folate | 12.54(9.15) | 12.452(4.06) | RIA | NR |
|  |  |  |  |  |  | Vitamin B12 | 485.49(287.93) | 515.44(195.62) | RIA | NR |
| Beydoun M et al. (B) | 2011 | United States of America | 45.6(0.7) | 1606(0/1606) | NCEP-ATP III | Hcy | 8(4.53) | 7.4(3.3) | CLIA | NR |
|  |  |  |  |  |  | Folate | 13.904(5.99) | 12.54(8.98) | RIA | NR |
|  |  |  |  |  |  | Vitamin B12 | 501.75(325.88) | 504.87(284.06) | RIA | NR |
| Garcin JM et al. | 2006 | France | 37.7(8.7) | 2045(2045/0) | IDF | Hcy | 11.3(6) | 10.9(5) | HPLC | NR |
| Nabipour I et al. | 2009 | Iran | ≥25 | 890(890/0) | NCEP-ATP III | Hcy | NR | NR | ELISA | 1(0.97–1.03) |
| Wang X et al. | 2014 | China | 61.40(11.4) | 1499(630/869) | CDS | Hcy | NR | NR | ECLIA | 1.75 (1.22–2.51) |
| Lee HS et al. | 2021 | South Korea | 58 (52.67) | 5902(2773/3129 | NCEP-ATP III | Hcy | NR | NR | FPI | 1.06 (1.05–1.07) |
| Rhee EJ et al. | 2007 | South Korea | 58.6(6.5) | 722(284/438) | NCEP-ATP III | Hcy | 9.7(3.08) | 9.43(4.41) | FPI | NR |
| Balcioğlu AS et al. | 2014 | Turkey | 59.6(14) | 150(60/90) | IDF | Hcy | 21.6(6.1) | 15.1(5.8) | FPI | NR |
| Shih Y et al. | 2023 | Taiwan | 64.8(8.77) | 396(164/232) | NCEP-ATP III | Hcy | 14.34(5.1) | 12.97(4.64) | FPI | NR |
| Feng SQ et al. | 2012 | China | 62.29(11.46) | 1680(709/971) | NCEP-ATP III | Hcy | NR | NR | ELISA | 1.37(1.00–1.89) |
| Vigna L et al. (A) | 2017 | Italy | 54(15) | 234(234/0) | NCEP-ATP III | Hcy | NR | NR | HPLC | 1.45(0.72–2.92) |
| Vigna L et al. (B) | 2017 | Italy | 54(14) | 591(0/591) | NCEP-ATP III | Hcy | NR | NR | HPLC | 0.96(0.63–1.46) |
| Butkowski E et al. | 2017 | Australia | 66(11) | 266(NR/NR) | NCEP-ATP III | Hcy | 10.6(3) | 9.5(2) | ECLIA | NR |
| Agoşton-Coldea L et al. | 2010 | Romania | 63.6(9.2) | 104(47/57) | NCEP-ATP III | Hcy | 14.8 (4.6) | 17.9 (10) | FPI | NR |
| Saeed A et al. | 2020 | Saudi Arabia | 53(14) | 446(218/228) | IDF | Hcy | NR | NR | FPI | 1.018 (1.011–1.025) |
| Liu C et al. | 2022 | China | 72 (6.6) | 3675(1617/2058) | IDF | Hcy | NR | NR | HPLC | 1.11 (0.86–1.43) |
| Kim SH et al. | 2011 | South Korea | 57.3(8.9) | 180(54/126) | NCEP-ATP III | Hcy | 11.5(3.7) | 9.2(2.7) | ELISA | NR |
| Yoon KS et al. | 2008 | South Korea | 54.6(9.3) | 230(68/162) | AHA/NHLBI | Hcy | 10.31(3.96) | 9.36(2.68) | ELISA | NR |
| Orluwene CG et al. | 2013 | Nigeria | 41(5.2) | 93(31/62) | NCEP-ATP III | Hcy | 17.5(3.2) | 9.8 (6.4) | HPLC | NR |
| Nayak B et al. (A) | 2013 | Trinidad and Tobago | 55.7(12.1) | 70(70/0) | IDF | Hcy | NR | NR | FPI | 0.527(0.116–2.39) |
| Nayak B et al. (B) | 2013 | Trinidad and Tobago | 54.3(11.7) | 112(0/112) | IDF | Hcy | NR | NR | FPI | 0.828(0.209–3.28) |
| Cankurtaran M et al. | 2006 | Turkey | 71.8(6.3) | 1255(466/789) | NCEP-ATP III | Hcy | NR | NR | FPI | 1.249 (0.920–1.69) |
| Herman W et al. | 2013 | Poland | 40–70 | 160(160/0) | IDF | Hcy | 11.52(6.87) | 10.08(5.44) | FPI | NR |
| Alihanoglu Y et al. | 2015 | Turkey | 55.5(9.6) | 72(40/32) | NCEP-ATP III | Hcy | 11.6(4) | 9.6(2.6) | ELISA | NR |
| Shin K et al. | 2012 | South Korea | 49.5(6.9) | 178(119/59) | NCEP-ATP III | Hcy | 12.44(5.31) | 10.7(3.38) | HPLC | NR |
| Khan R et al. | 2015 | United States of America | 54.87(12.84) | 5227(1909/3318) | NCEP-ATP III | Hcy | 9.94(6.37) | 9.15(3.44) | FPI | NR |
| Al-Daghri N et al. | 2007 | Saudi Arabia | 48.82(12.22) | 581(294/287) | IDF | Hcy | 9.81 (10.77) | 10.58 (9.67) | HPLC | NR |
| Huang Z et al. | 2019 | China | 53.41(7) | 484(484/0) | NCEP-ATP III | Hcy | NR | NR | HPLC | 1.904(1.073–3.378) |
|  |  |  |  |  |  | Vitamin B12 | 704.69(275.5) | 741.8(270.44) | ECLIA | NR |
| Yue C et al. | 2012 | China | 63(2.2) | 62(29/33) | WHO 1999 | Hcy | 16.23(6.87) | 12.11(2.07) | HPLC | NR |
| Xiang L et al. | 2016 | China | NR(NR) | 594(NR/NR) | NCEP-ATP III | Hcy | 12.2(7) | 12.6(8.4) | FPI | NR |
| Shu-Heng C et al. | 2020 | China | 20–74 | 8201(NR/NR) | NCEP-ATP III | Hcy | NR | NR | FPI | 1.16(1.02–1.31) |
| Di Cianni G et al. | 2007 | Italy | 34.7(4.2) | 166(0/166) | NCEP-ATP III | Hcy | 10.3(6.1) | 8.1(3.2) | CLIA | NR |
| Sengwenyo D et al. | 2013 | South Africa | 18–65 | 382(96/286) | NCEP-ATP III | Hcy | 10.68(3.433) | 9.23(2.924) | FPI | NR |
| Sung S et al. | 2019 | South Korea | 20–58 | 8606(8606/0) | IDF | Hcy | NR | NR | ELISA | 1.09(0.94–1.27) |
| Bauduceau B et al. | 2005 | France | 38.6(8.8) | 2045(2045/0) | NCEP-ATP III | Hcy | 11.31(5.91) | 10.87(4.43) | FPI | NR |
| Panagiotakos D et al. | 2004 | Greece | 45(13) | 2282(1128/1154) | NCEP-ATP III | Hcy | NR | NR | FPI | 1.021(0.981–1.06) |
| Prasad M et al. | 2014 | United States of America | 43(10) | 1000(701/299) | AHA/NHLBI | Hcy | NR | NR | HEI | 1.6(1.1–2.32) |
| Shin YR et al. | 2012 | South Korea | 65.7(8) | 74(31/43) | NCEP-ATP III | Hcy | 12(5.2) | 11(5.1) | HPLC | NR |
| Mohorko N et al. | 2015 | Slovenia | 25–49 | 61(21/40) | AHA/NHLBI | Hcy | 10.4(2.5) | 10.8(2.6) | FPI | NR |
| Yu S et al. | 2020 | China | 54.42(10.73) | 6837(3150/3687) | NCEP-ATP III | Hcy | NR | NR | FPI | 0.9(0.81–0.99) |
| Hu F et al. | 2022 | China | 63.76(9.35) | 7035(2505/4530) | CDS | Hcy | 12.375(1.74) | 12.53(1.73) | HPLC | NR |
| Baltaci D et al.(A) | 2013 | Turkey | 19–53 | 414(NR/NR) | NCEP-ATP III | Folate | 8.5(3.8) | 8.3(3.7) | CLIA | NR |
|  |  |  |  |  |  | Vitamin B12 | 241.4(83.2) | 238.43(94.2) | CLIA | NR |
| Baltaci D et al.(B) | 2012 | Turkey | 37.7(9.1) | 184(0/184) | NCEP-ATP III | Folate | 9.1(7.8) | 6.8(3.3) | CLIA | NR |
|  |  |  |  |  |  | Vitamin B12 | 245.1(145.3) | 336.2 (163.1) | CLIA | NR |
| Chen AR et al. | 2010 | China | 58.85(11.52) | 173(96/77) | CDS | Folate | 14.28(10) | 19.7(10.7) | RIA | NR |
|  |  |  |  |  |  | Vitamin B12 | 506.08(249.57) | 633.3(298.44) | RIA | NR |
| Kulaksizoglu B et al. | 2016 | Turkey | 38.25(7.69) | 64(36/28) | IDF | Folate | 8.85(3.3) | 9(2.58) | CLIA | NR |
|  |  |  |  |  |  | Vitamin B12 | 186.08(85.76) | 254.22(116.38) | CLIA | NR |
| Kanagasabai T et al. | 2019 | United States of America | 47.6–54.4 | 2049(1084/965) | IDF | Folate | NR | NR | CLIA | 0.55 (0.11–2.75) |
|  |  |  |  |  |  | Vitamin B12 | NR | NR | CLIA | 2.45 (1.09–5.5) |
| Guarnizo-Poma M et al. | 2018 | Peru | 38.3(10.8) | 346(117/229) | AHA/NHLBI | Vitamin B12 | 461.63(187.34) | 515.98(212.43) | ECLIA | NR |
| Ashok P et al. | 2022 | India | 35–64 | 300(0/300) | IDF | Hcy | NR | NR | CLIA | 3.33(1.52–7.29) |
|  |  |  |  |  |  | Folate | NR | NR | RIA | 0.84(0.39–1.80) |
|  |  |  |  |  |  | Vitamin B12 | NR | NR | RIA | 0.6(0.28–1.28) |
| Koo Y-S et al. | 2022 | South Korea | 35.9 (0.3) | 1730(0/1730) | NCEP-ATP III | Folate | NR | NR | CLIA | 1.35 (0.89–2.05) |
| Mba C et al. | 2022 | Cameroon | 38.2(8.6) | 578(208/370) | AHA/NHLBI | Folate | NR | NR | HPLC | 0.74(0.6–0.9) |
| Maiti S et al. | 2015 | India | 46.2(5.3) | 200(66/134) | IDF | Vitamin B12 | 161(97.3) | 312.88(119.7) | CLIA | NR |
| Villatoro-Santos C et al. | 2020 | Mesoamerican countries | 38.7(7.5) | 524(260/264) | NCEP-ATP III | Hcy | NR | NR | CLIA | 0.85(0.56–1.29) |
|  |  |  |  |  |  | Vitamin B12 | NR | NR | CLIA | 1.17(0.74–1.86) |

**Table S3. Criteria for clinical diagnosis of metabolic syndrome used in the included studies**

| **Diagnostic criteria** | **Definition** |
| --- | --- |
| **American Heart Association/National Heart Lung and Blood Institute (AHA/NHLBI)** | Three of more of following five criteria needed to be present: (1) waist circumference > 90 cm for men and >80 cm for women, (2) fasting serum triglycerides >= 150 mg/dl or on drug treatment for elevated triglycerides, (3) HDL cholesterol <40 mg/dl for men and <50 mg/dl for women or on drug treatment to increase HDL cholesterol, (4) fasting blood glucose >100 mg/dl or on drug treatment for diabetes and (5) systolic blood pressure >130 mmHg or diatolic > 85 mmHg or on drug treatment for hypertension. |
| **National Cholesterol Education Program-Adult Treatment Panel III(NCEP-ATP III)** | Three of the following factors: waist circumference >102cm (men) or >88cm(women), triglyceride level ≥1.69mmol/l, HDL cholesterol<1.03mmol/l (men) or < 1.29mmol/l (women), blood pressure ≥130/85mmHg, or fasting glucose ≥6.1mmol/l. |
| **International Diabetes Federation(IDF)** | Central obesity plus any two of the following four factors: raised fasting plasma glucose (≥100 mg/dL) or treatment of previously diagnosed type 2 diabetes, raised blood pressure (systolic blood pressure ≥130 or diastolic blood pressure ≥85 mmHg) or treatment of previously diagnosed hypertension, raised triglycerides (≥150 mg/dl), and reduced HDL cholesterol (<40 mg/dL in males, <50 mg/dL in females). |
| **Chinese Diabetes Society(CDS)** | Three of the following 5 criteria: abdominal obesity (waistline ≥90 cm in adult male and 85 cm in adult female subjects), elevated blood pressure (SBP ≥130 mmHg and/or DBP ≥85 mmHg, or on treatment for diagnosed hypertension formerly), hyperglycemia (FBG ≥6.1 mmol/L, or on appropriate hypoglycemic medication), elevated TG (≥1.7 mmol/L, or on appropriate lipid-lowering medication) and reduced HDL-C (<1.03 mmol/L in adult male and <1.30 mmol/L in adult female subjects, or on appropriate lipid-lowering medication). |
| **World Health Organization(WHO)** | Impaired glucose intolerance, Impaired fasting glycemiaI, T2DM, or lowered insulin sensitivity plus any 2 of the following: (1)Men: waist-to-hip ratio >0.90,women: waist-to-hip ratio >0.85 and/or BMI >30; (2) TG ≥150 mg/dL and/or HDL-C <35 mg/dL in men or <39 mg/dL in women; systolic blood pressure ≥140 mmHg or diatolic ≥ 90 mmHg and (3) microalbuminuria |

**Table S4. Quality assessment of included studies**

|  | **NEWCASTLE - OTTAWA QUALITY ASSESSMENT SCALE FOR COHORT STUDIES** | | | | | | | | | | |
| --- | --- | --- | --- | --- | --- | --- | --- | --- | --- | --- | --- |
| **STUDY** | | **SELECTION** | | | | **COMPARABILITY** | **OUTCOME** | |  |  |  |
|  | | **Representativeness of the exposed cohort** | **Selection of the non-exposed cohort** | **Ascertainment of exposure** | **Demonstration that outcome of interest was not present at start of study** | **Comparability of Cohorts on the Basis of the Design or Analysis Maximum : ☆☆** | **Assessment of outcome** | **Was follow-up long enough for outcomes to occur** | **Adequacy of follow up of cohorts** | **SCORE** | **Evidence quality** |
| **Zhu J et al.** | | ☆ | ☆ | ☆ | ☆ | ☆☆ | ☆ | ☆ | ☆ | 9 | Low risk of bias |
| **Hajer G et al.** | | ☆ | ☆ | ☆ | ☆ | ☆☆ | ☆ | ☆ | ☆ | 9 | Low risk of bias |
| **Garcin JM et al.** | | ☆ | ☆ | ☆ | ☆ | ☆ | ☆ | ☆ | ☆ | 8 | Low risk of bias |
| **Wang X et al.** | | ☆ | ☆ | ☆ | ☆ | ☆☆ | ☆ | ☆ | ☆ | 9 | Low risk of bias |
| **Lee HS et al.** | | ☆ | ☆ | ☆ | ☆ | ☆ | ☆ | ☆ | ☆ | 8 | Low risk of bias |
| **Vigna L et al. (A)** | | ☆ | ☆ | ☆ | ☆ | ☆ | ☆ | ☆ | ☆ | 8 | Low risk of bias |
| **Vigna L et al. (B)** | | ☆ | ☆ | ☆ | ☆ | ☆ | ☆ | ☆ | ☆ | 8 | Low risk of bias |
| **Liu C et al.** | | ☆ | ☆ | ☆ | ☆ | ☆☆ | ☆ | ☆ | ☆ | 9 | Low risk of bias |
| **Di Cianni G et al.** | | ☆ | ☆ | ☆ | ☆ | ☆ | ☆ | ☆ | ☆ | 8 | Low risk of bias |

|  | |  | | **NEWCASTLE - OTTAWA QUALITY ASSESSMENT SCALE FOR CASE-CONTROL STUDIES** | | | | | | | | | |
| --- | --- | --- | --- | --- | --- | --- | --- | --- | --- | --- | --- | --- | --- |
| **STUDY** | | **SELECTION** | | | | **COMPARABILITY** | | **EXPOSURE** | |  |  |  |  |
|  | | **Is the case definition adequate?** | | **Representativeness of the cases** | **Selection of Controls** | **Definition of Controls** | **Comparability of cases and controls on the basis of the design or analysis (Maximum : ☆☆ )** |  | **Same method of ascertainment for cases and controls** | **Non-Response rate** | **Ascertainment of exposure** | **SCORE** | **Evidence quality** |
| **Narang M et al.** | | ☆ | | ☆ | ☆ | ☆ | ☆ | | ☆ | ☆ | ☆ | 8 | Low risk of bias |
| **Guven A et al.** | | ☆ | | ☆ |  |  | ☆ | | ☆ | ☆ | ☆ | 6 | High risk of bias |
| **Vaya A et al.** | | ☆ | | ☆ | ☆ | ☆ | ☆☆ | | ☆ | ☆ | ☆ | 9 | Low risk of bias |
| **Kang J et al.** | | ☆ | | ☆ | ☆ | ☆ | ☆ | | ☆ | ☆ | ☆ | 8 | Low risk of bias |
| **Wang J et al.** | | ☆ | | ☆ | ☆ | ☆ | ☆ | | ☆ | ☆ | ☆ | 8 | Low risk of bias |
| **Balcioğlu AS et al.** | | ☆ | | ☆ | ☆ | ☆ | ☆☆ | | ☆ | ☆ | ☆ | 9 | Low risk of bias |
| **Butkowski E et al.** | | ☆ | | ☆ | ☆ | ☆ | ☆ | | ☆ | ☆ | ☆ | 8 | Low risk of bias |
| **Yoon KS et al.** | | ☆ | | ☆ | ☆ | ☆ | ☆☆ | | ☆ | ☆ | ☆ | 9 | Low risk of bias |
| **Orluwene CG et al.** | | ☆ | | ☆ |  |  | ☆ | | ☆ | ☆ | ☆ | 6 | High risk of bias |
| **Herman W et al.** | | ☆ | | ☆ | ☆ | ☆ | ☆☆ | | ☆ | ☆ | ☆ | 9 | Low risk of bias |
| **Yue C et al.** | | ☆ | | ☆ | ☆ | ☆ | ☆☆ | | ☆ | ☆ | ☆ | 9 | Low risk of bias |
| **Baltaci D et al.(B)** | | ☆ | | ☆ | ☆ | ☆ | ☆ | | ☆ | ☆ | ☆ | 8 | Low risk of bias |
| **Kulaksizoglu B et al.** | | ☆ | | ☆ | ☆ | ☆ | ☆☆ | | ☆ | ☆ | ☆ | 9 | Low risk of bias |

|  | **NEWCASTLE - OTTAWA QUALITY ASSESSMENT SCALE FOR CROSS-SECTIONAL STUDIES** | | | | | | | | | |
| --- | --- | --- | --- | --- | --- | --- | --- | --- | --- | --- |
| **STUDY** | | **SELECTION** | | | | **COMPARABILITY** | **OUTCOME** | |  |  |
|  | | **Representativeness of the sample** | **Sample size** | **Non-respondents** | **Ascertainment of the exposure (risk factor)** | **The subjects in different outcome groups are comparable, based on the study design or analysis. Confounding factors are controlled.**  **Maximum : ☆☆** | **Assessment of outcome** | **Statistical test** | **SCORE** | **Evidence quality** |
| **Catena C et al.** | | ☆ | ☆ | ☆ | ☆ | ☆☆ | ☆ | ☆ | *8* | Low Risk of Bias |
| **Sempértegui F et al.** | | ☆ | ☆ | ☆ | ☆ | ☆☆ | ☆ | ☆ | *8* | Low Risk of Bias |
| **Sreckovic B et al. (A)** | | ☆ | ☆ | ☆ | ☆ | ☆☆ | ☆ | ☆ | *8* | Low Risk of Bias |
| **Sreckovic B et al. (B)** | | ☆ | ☆ | ☆ | ☆ | ☆☆ | ☆ | ☆ | *8* | Low Risk of Bias |
| **Loprinzi P et al. (A)** | | ☆ | ☆ | ☆ | ☆ | ☆ | ☆ | ☆ | *7* | Low Risk of Bias |
| **Loprinzi P et al. (B)** | | ☆ | ☆ | ☆ | ☆ | ☆ | ☆ | ☆ | *7* | Low Risk of Bias |
| **Fakhrzadeh H et al. (A)** | | ☆ | ☆ | ☆ | ☆ | ☆ | ☆ |  | *6* | High Risk of Bias |
| **Nurjahan F et al.** | | ☆ | ☆ | ☆ | ☆ | ☆ | ☆ |  | *6* | High Risk of Bias |
| **Akter K et al.** | | ☆ | ☆ | ☆ | ☆ | ☆ | ☆ |  | *6* | High Risk of Bias |
| **Azarpazhooh A et al.** | | ☆ | ☆ | ☆ | ☆ | ☆ | ☆ |  | *6* | High Risk of Bias |
| **Fakhrzadeh H et al. (B)** | | ☆ | ☆ | ☆ | ☆ | ☆ | ☆ |  | *6* | High Risk of Bias |
| **Beydoun M et al. (A)** | | ☆ | ☆ | ☆ | ☆ | ☆☆ | ☆ | ☆ | *8* | Low Risk of Bias |
| **Beydoun M et al. (B)** | | ☆ | ☆ | ☆ | ☆ | ☆☆ | ☆ | ☆ | *8* | Low Risk of Bias |
| **Nabipour I et al.** | | ☆ | ☆ | ☆ | ☆ | ☆☆ | ☆ | ☆ | *8* | Low Risk of Bias |
| **Rhee EJ et al.** | | ☆ | ☆ | ☆ | ☆ | ☆☆ | ☆ | ☆ | *8* | Low Risk of Bias |
| **Shih Y et al.** | | ☆ | ☆ | ☆ | ☆ | ☆ | ☆ | ☆ | *7* | Low Risk of Bias |
| **Feng SQ et al.** | | ☆ | ☆ | ☆ | ☆ | ☆☆ | ☆ | ☆ | *8* | Low Risk of Bias |
| **Agoşton-Coldea L et al.** | | ☆ | ☆ | ☆ | ☆ | ☆ | ☆ | ☆ | *7* | Low Risk of Bias |
| **Saeed A et al.** | | ☆ | ☆ | ☆ | ☆ | ☆☆ | ☆ | ☆ | *8* | Low Risk of Bias |
| **Kim SH et al.** | | ☆ | ☆ | ☆ | ☆ | ☆ | ☆ | ☆ | *7* | Low Risk of Bias |
| **Nayak B et al. (A)** | | ☆ | ☆ | ☆ | ☆ | ☆☆ | ☆ | ☆ | *8* | Low Risk of Bias |
| **Nayak B et al. (B)** | | ☆ | ☆ | ☆ | ☆ | ☆☆ | ☆ | ☆ | *8* | Low Risk of Bias |
| **Cankurtaran M et al.** | | ☆ | ☆ | ☆ | ☆ | ☆ | ☆ | ☆ | *7* | Low Risk of Bias |
| **Alihanoglu Y et al.** | | ☆ | ☆ | ☆ | ☆ | ☆☆ | ☆ | ☆ | *8* | Low Risk of Bias |
| **Shin K et al.** | | ☆ | ☆ | ☆ | ☆ | ☆ | ☆ | ☆ | *7* | Low Risk of Bias |
| **Khan R et al.** | | ☆ | ☆ | ☆ | ☆ | ☆☆ | ☆ | ☆ | *8* | Low Risk of Bias |
| **Al-Daghri N et al.** | | ☆ | ☆ | ☆ | ☆ | ☆ | ☆ | ☆ | *7* | Low Risk of Bias |
| **Huang Z et al.** | | ☆ | ☆ | ☆ | ☆ | ☆☆ | ☆ | ☆ | *8* | Low Risk of Bias |
| **Xiang L et al.** | | ☆ | ☆ | ☆ | ☆ | ☆ | ☆ | ☆ | *7* | Low Risk of Bias |
| **Shu-Heng C et al.** | | ☆ | ☆ | ☆ | ☆ | ☆☆ | ☆ | ☆ | *8* | Low Risk of Bias |
| **Sengwenyo D et al.** | | ☆ | ☆ | ☆ | ☆ | ☆ | ☆ | ☆ | *7* | Low Risk of Bias |
| **Sung S et al.** | | ☆ | ☆ | ☆ | ☆ | ☆☆ | ☆ | ☆ | *8* | Low Risk of Bias |
| **Bauduceau B et al.** | | ☆ | ☆ | ☆ | ☆ | ☆ | ☆ | ☆ | *7* | Low Risk of Bias |
| **Panagiotakos D et al.** | | ☆ | ☆ | ☆ | ☆ | ☆☆ | ☆ | ☆ | *8* | Low Risk of Bias |
| **Prasad M et al.** | | ☆ | ☆ | ☆ | ☆ | ☆ | ☆ | ☆ | *7* | Low Risk of Bias |
| **Shin YR et al.** | | ☆ | ☆ | ☆ | ☆ | ☆☆ | ☆ | ☆ | *8* | Low Risk of Bias |
| **Mohorko N et al.** | | ☆ | ☆ | ☆ | ☆ | ☆ | ☆ | ☆ | *7* | Low Risk of Bias |
| **Yu S et al.** | | ☆ | ☆ | ☆ | ☆ | ☆ | ☆ | ☆ | *7* | Low Risk of Bias |
| **Hu F et al.** | | ☆ | ☆ | ☆ | ☆ | ☆☆ | ☆ | ☆ | *8* | Low Risk of Bias |
| **Baltaci D et al.(A)** | | ☆ | ☆ | ☆ | ☆ | ☆ | ☆ | ☆ | *7* | Low Risk of Bias |
| **Chen AR et al.** | | ☆ | ☆ | ☆ | ☆ | ☆☆ | ☆ | ☆ | *8* | Low Risk of Bias |
| **Kanagasabai T et al.** | | ☆ | ☆ | ☆ | ☆ | ☆ | ☆ | ☆ | *7* | Low Risk of Bias |
| **Guarnizo-Poma M et al.** | | ☆ | ☆ | ☆ | ☆ | ☆☆ | ☆ | ☆ | *8* | Low Risk of Bias |
| **Ashok P et al.** | | ☆ | ☆ | ☆ | ☆ | ☆ | ☆ | ☆ | *7* | Low Risk of Bias |
| **Koo Y-S et al.** | | ☆ | ☆ | ☆ | ☆ | ☆☆ | ☆ | ☆ | *8* | Low Risk of Bias |
| **Mba C et al.** | | ☆ | ☆ | ☆ | ☆ | ☆ | ☆ | ☆ | *7* | Low Risk of Bias |
| **Villatoro-Santos C et al.** | | ☆ | ☆ | ☆ | ☆ | ☆ | ☆ | ☆ | *7* | Low Risk of Bias |
| **Maiti S et al.** | | ☆ | ☆ | ☆ | ☆ | ☆☆ | ☆ | ☆ | *8* | Low Risk of Bias |

**Figure S1. Subgroup analysis according to continents of the association between vitamin B12 and risk of MetS**

**
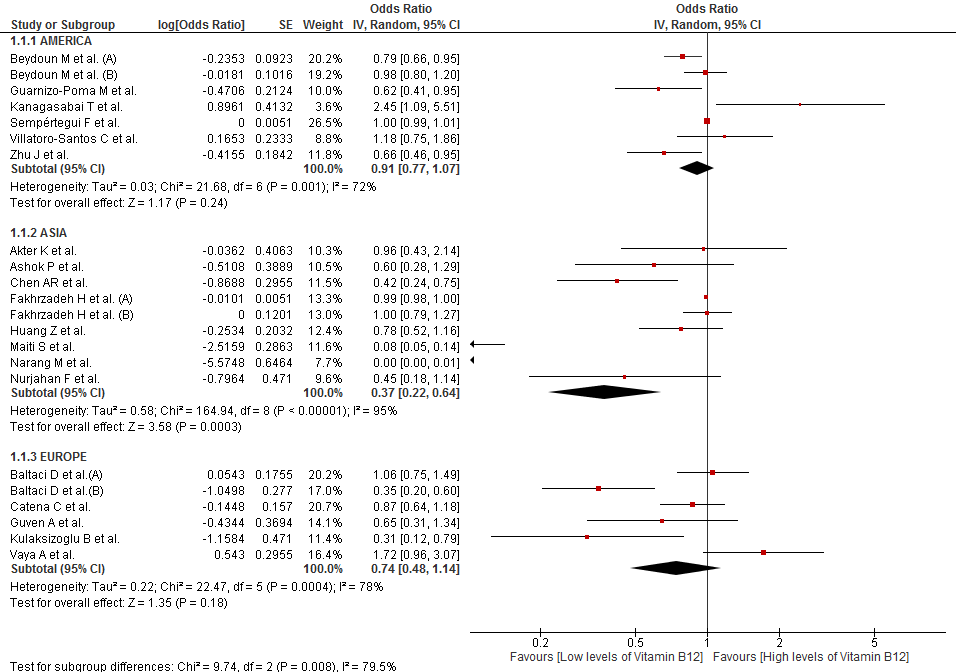
**

**Figure S2. Subgroup analysis according to gender of the association between vitamin B12 and risk of MetS**

**
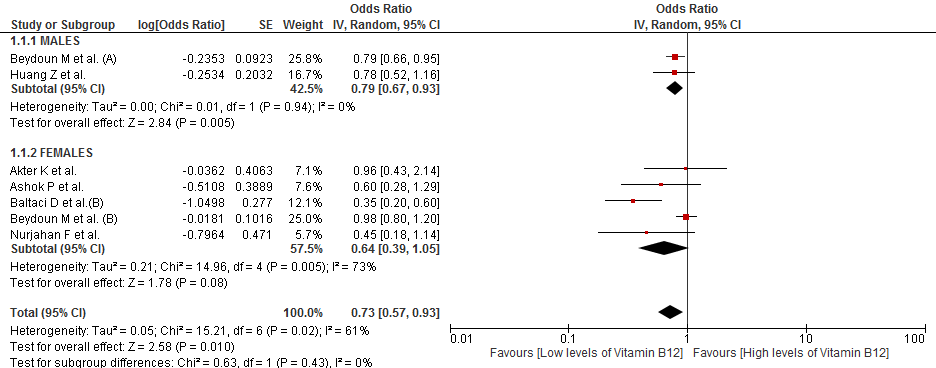
**

**Figure S3. Subgroup analysis according to assay method of the association between vitamin B12 and risk of MetS**

**
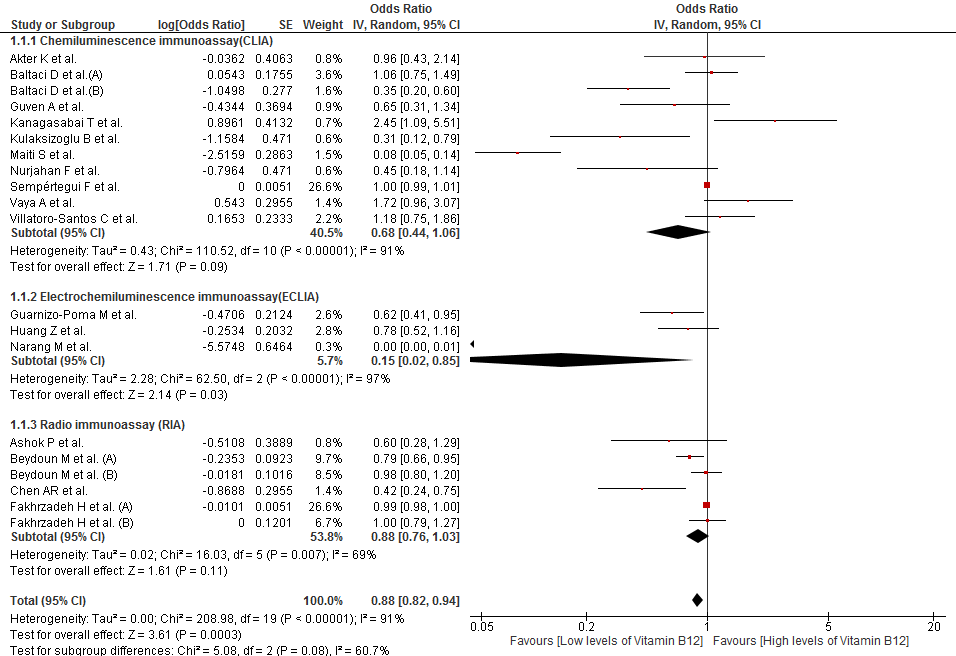
**

**Figure S4. Subgroup analysis according to diagnostic criteria of the association between vitamin B12 and risk of MetS**

**
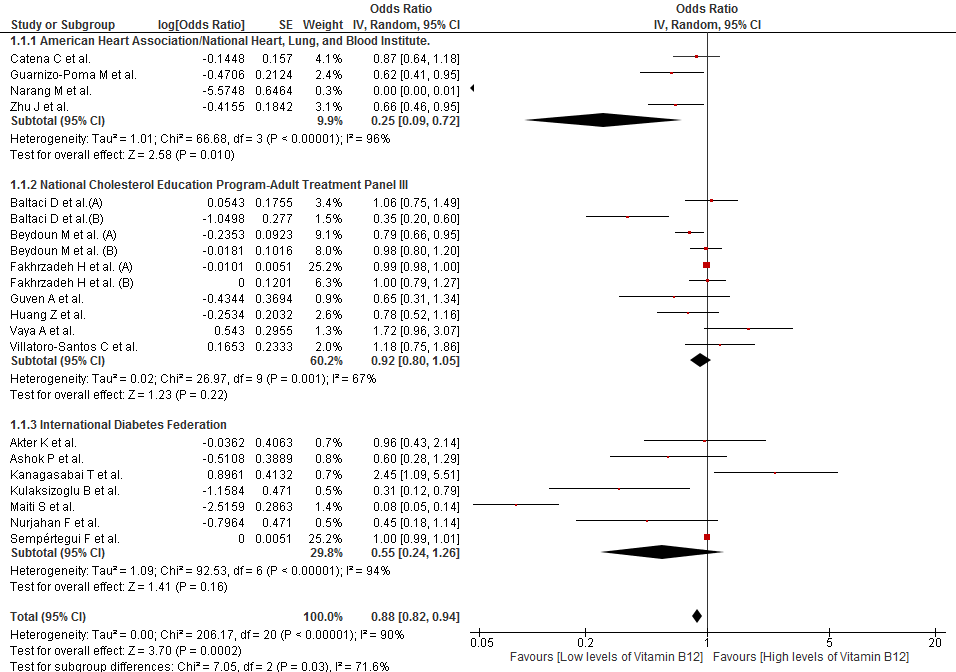
**

**Figure S5. Sensitivity analysis according to risk of bias of the association between vitamin B12 levels and MetS**

**
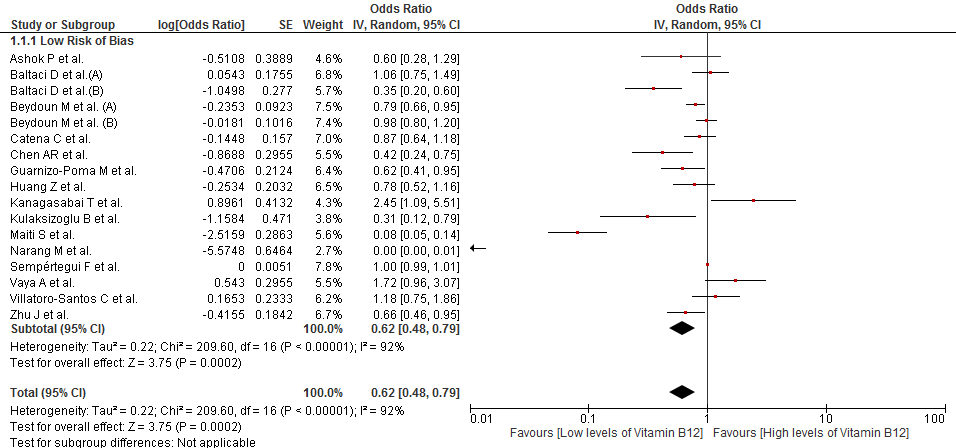
**

**Figure S6. Subgroup analysis according to continents of the association between folate and risk of MetS**

**
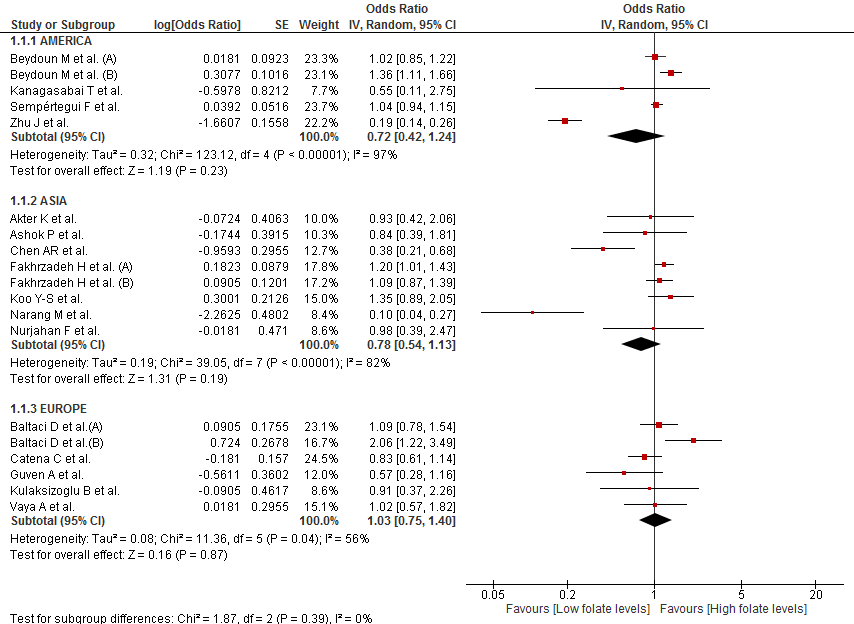
**

**Figure S7. Subgroup analysis according to gender of the association between folate and risk of MetS**

**
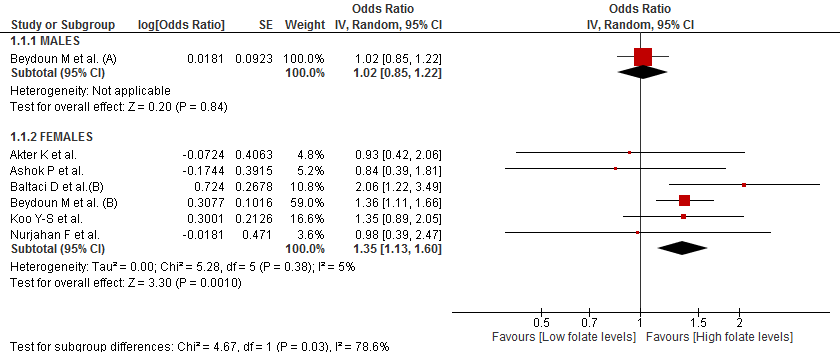
**

**Figure S8. Subgroup analysis according to assay method of the association between folate and risk of MetS**

**
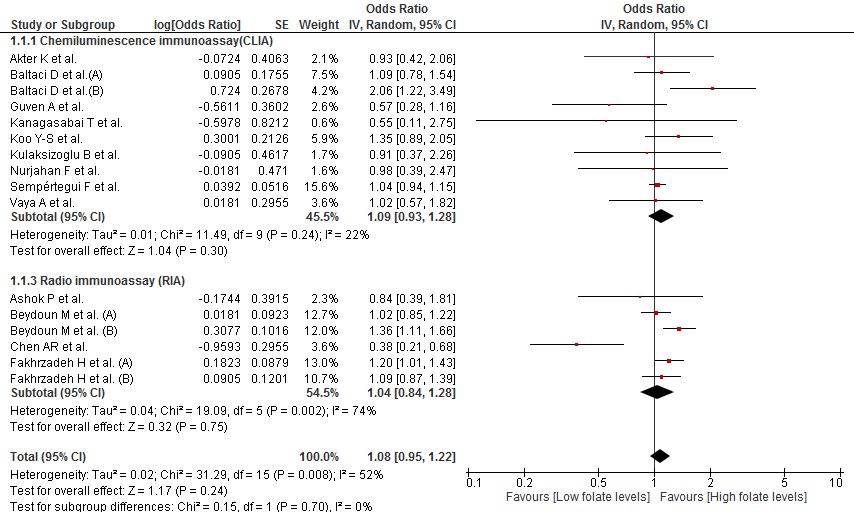
**

**Figure S9. Subgroup analysis according to diagnostic criteria of the association between folate and risk of MetS**

**
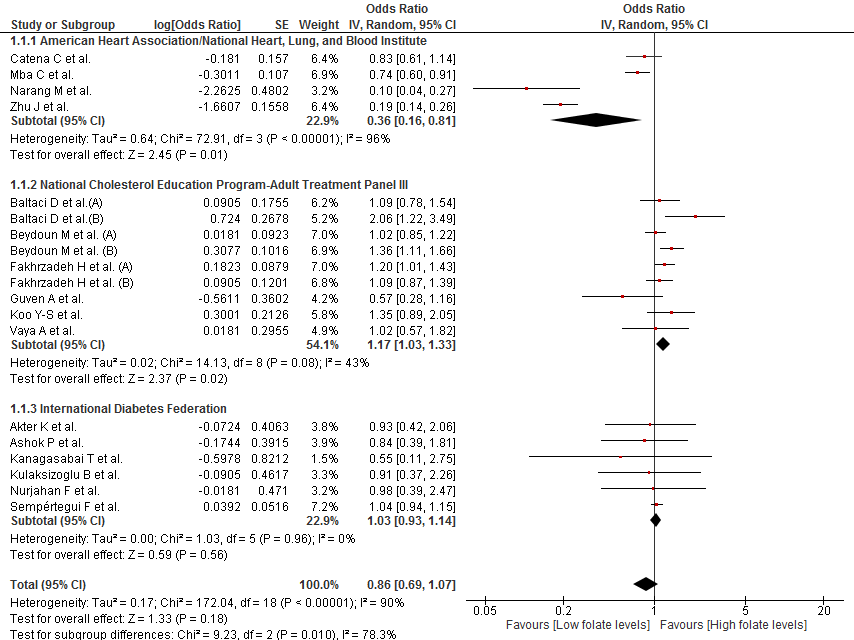
**

**Figure S10. Sensitivity analysis according to risk of bias of the association between folate levels and MetS**

**
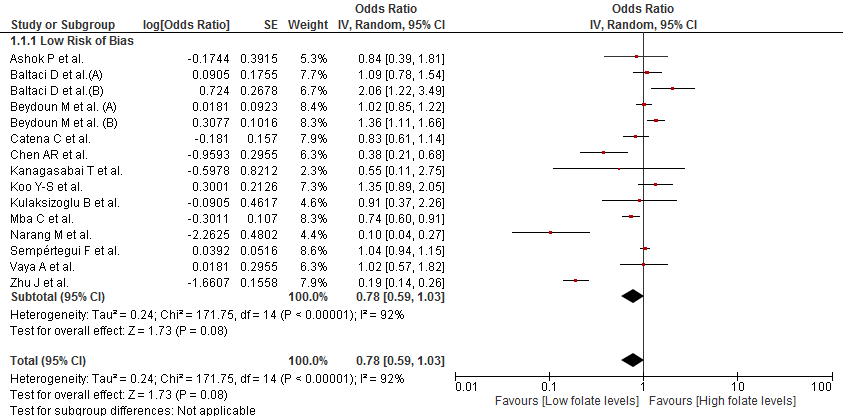
**

**Figure S11. Subgroup analysis according to continents of the association between homocysteine and risk of MetS**

**
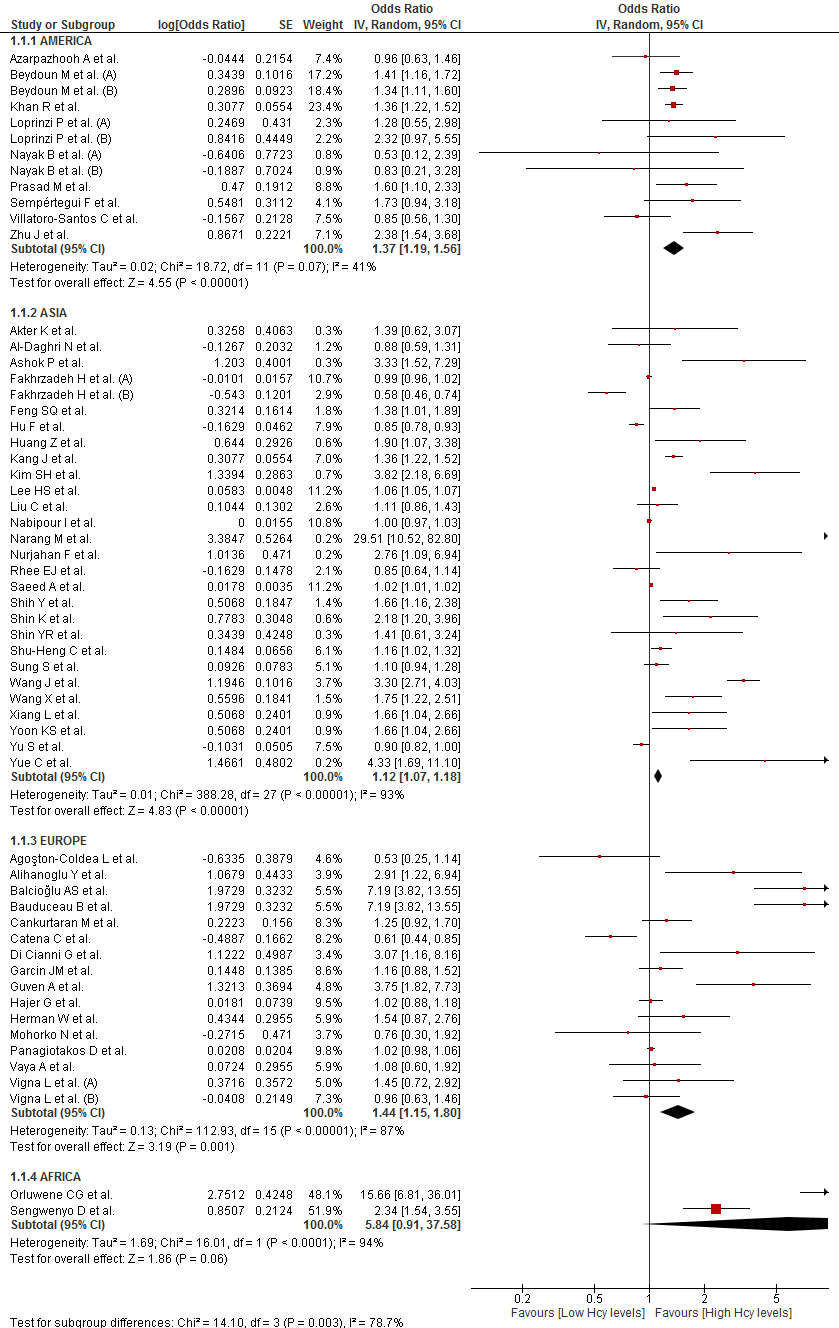
**

**Figure S12. Subgroup analysis according to gender of the association between homocysteine and risk of MetS**

**
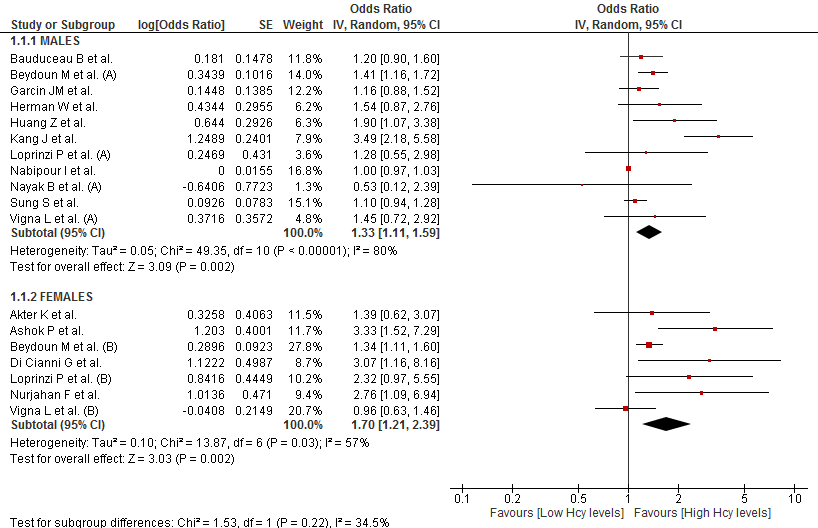
**

**Figure S13. Subgroup analysis according to assay method of the association between homocysteine and risk of MetS**

**
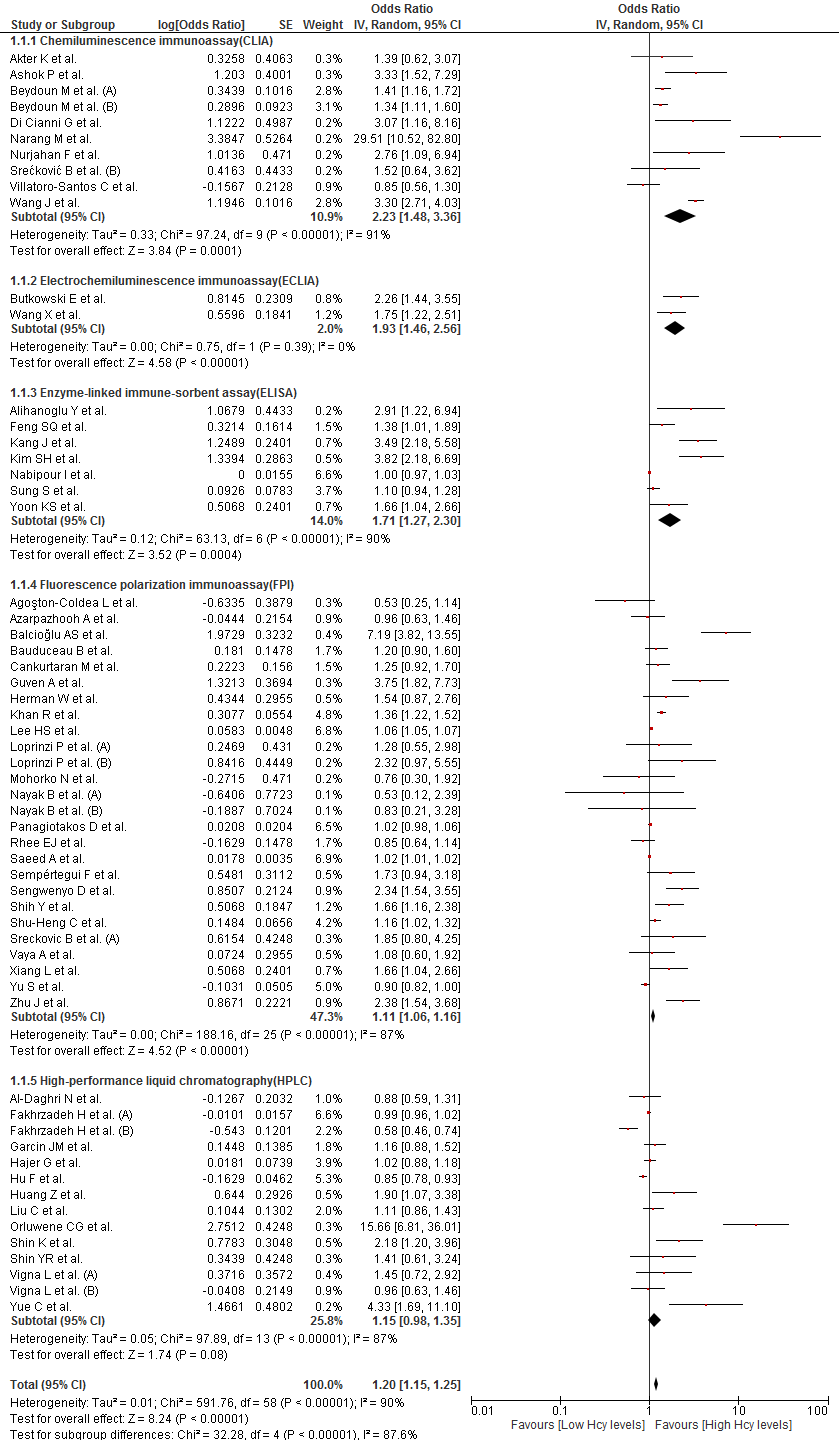
**

**Figure S14. Subgroup analysis according to diagnostic criteria of the association between homocysteine and risk of MetS**

**
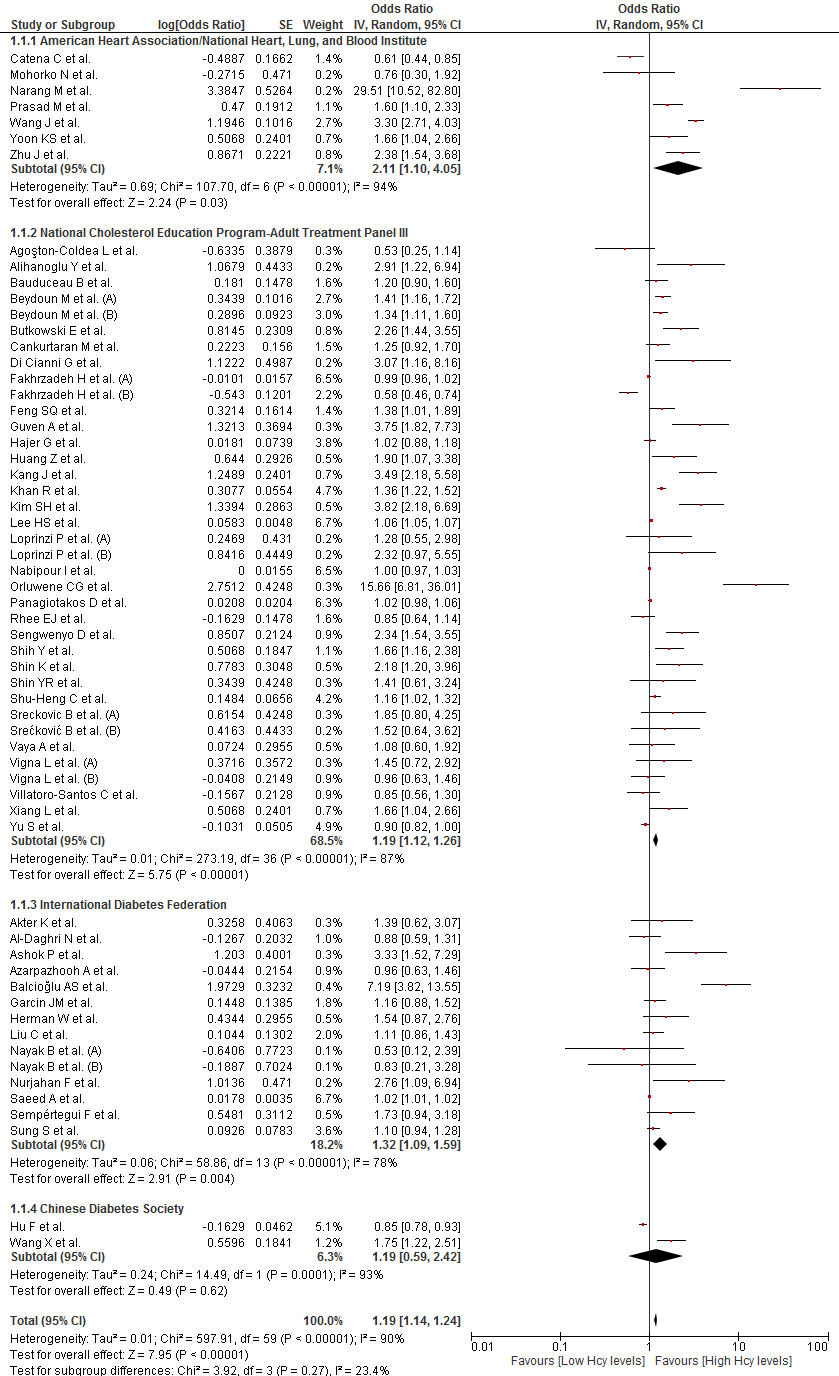
**

**Figure S15. Sensitivity analysis according to risk of bias of the association between Hcy levels and MetS**

**
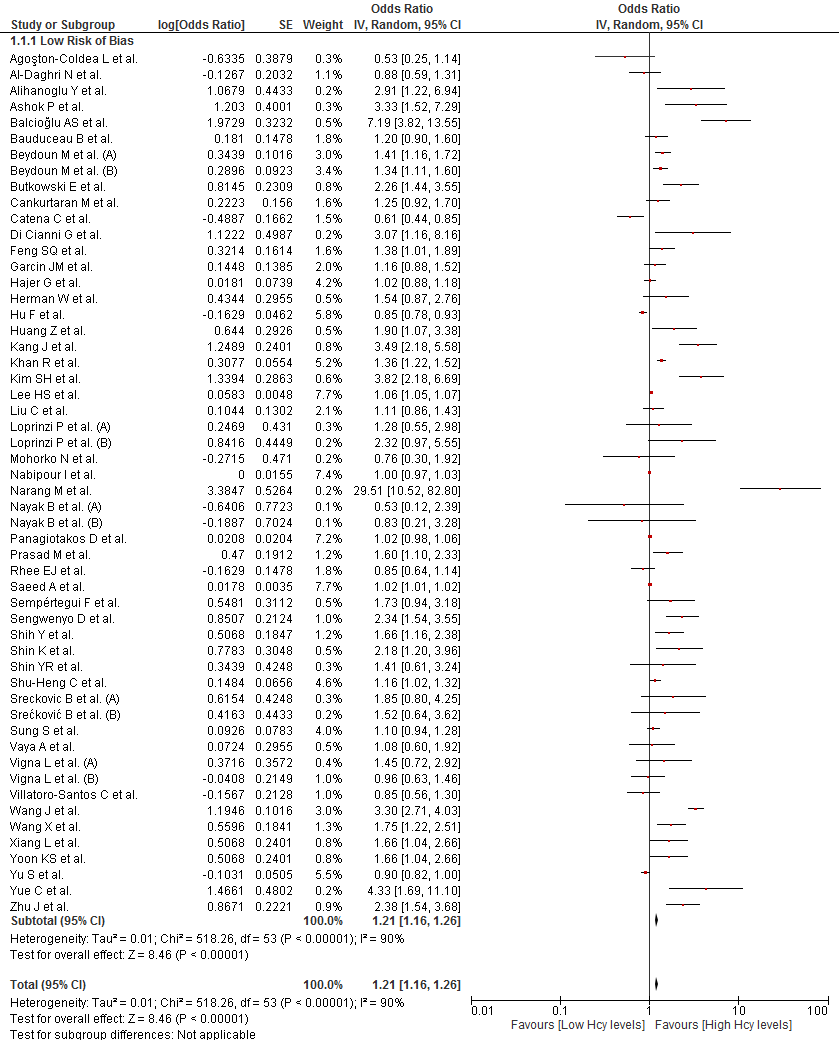
**

**Figure S16. Trim and Fill method of all the studies that evaluated the association between Hcy and MetS**


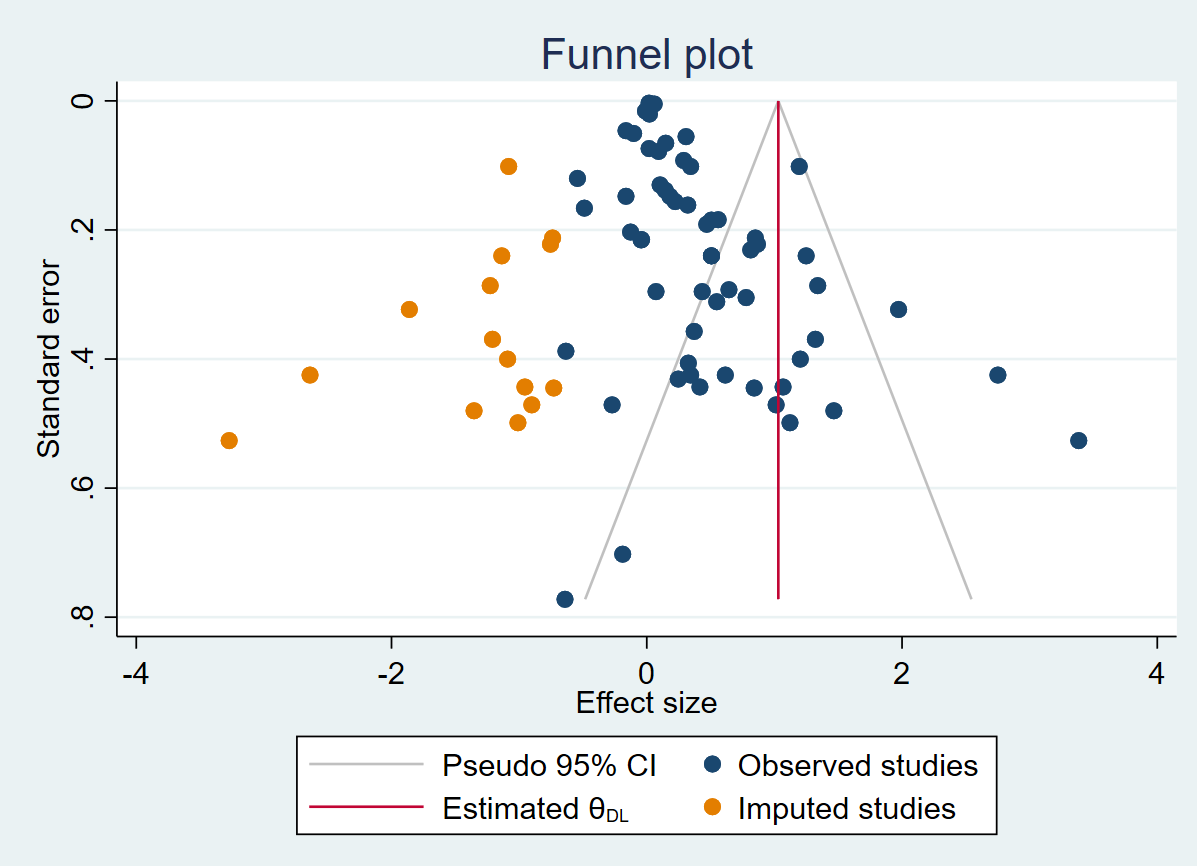

Supplement: Supplementary file 1 [file DataSheet_1.docx]
